# Predicting Surgical Difficulty in Rectal Cancer Surgery: A Systematic Review of Artificial Intelligence Models Applied to Pre-Operative MRI

**DOI:** 10.3390/cancers17050812

**Published:** 2025-02-26

**Authors:** Conor Hardacre, Thomas Hibbs, Matthew Fok, Rebecca Wiles, Nada Bashar, Shakil Ahmed, Miguel Mascarenhas Saraiva, Yalin Zheng, Muhammad Ahsan Javed

**Affiliations:** 1 University Hospitals of Liverpool Group, Liverpool L7 8YE, UKnada.bashar@liverpoolft.nhs.uk (N.B.); ahsanj@liverpool.ac.uk (M.A.J.); 2 Faculty of Health and Life Sciences, University of Liverpool, Liverpool L69 7ZX, UK; yzheng@liverpool.ac.uk; 3 Precision Medicine Unit, Department of Gastroenterology, São João University Hospital, 4200-427 Porto, Portugal; miguelmascarenhassaraiva@gmail.com; 4 Institute of Systems, Molecular and Integrative Biology, University of Liverpool, Liverpool L69 7TX, UK

**Keywords:** rectal cancer surgery, rectal adenocarcinoma, difficulty prediction, artificial intelligence, machine learning, radiology, MRI

## Abstract

**Introduction:** Following the rapid advances in minimally invasive surgery, there are a multitude of surgical modalities available for resecting rectal cancers. Robotic resections represent the current pinnacle of surgical approaches. Currently, decisions on the surgical modality depend on local resources and the expertise of the surgical team. Given limited access to robotic surgery, developing tools based on pre-operative data that can predict the difficulty of surgery would streamline the efficient utilisation of resources. This systematic review aims to appraise the existing literature on artificial intelligence (AI)-driven preoperative MRI analysis for surgical difficulty prediction to identify knowledge gaps and promising models warranting further clinical evaluation. **Methods:** A systematic review and narrative synthesis were undertaken in accordance with PRISMA and SWiM guidelines. Systematic searches were performed on Medline, Embase, and the CENTRAL Trials register. Studies published between 2012 and 2024 were included where AI was applied to preoperative MRI imaging of adult rectal cancer patients undergoing surgeries, of any approach, for the purpose of stratifying surgical difficulty. Data were extracted according to a pre-specified protocol to capture study characteristics and AI design; the objectives and performance outcome metrics were summarised. **Results:** Systematic database searches returned 568 articles, 40 ultimately included in this review. AI to support preoperative difficulty assessments were identified across eight domains (direct surgical difficulty grading, extramural vascular invasion (EMVI), lymph node metastasis (LNM), lymphovascular invasion (LVI), perineural invasion (PNI), T staging, and the requirement for multiple linear stapler firings. For each, at least one model was identified with very good performance (AUC scores of >0.80), with several showing excellent performance considerably above this threshold. **Conclusions:** AI tools applied to preoperative rectal MRI to support preoperative difficulty assessment for rectal cancer surgeries are emerging, with the progressing development and strong performance of many promising models. These warrant further clinical evaluation, which can aid personalised surgical approaches and ensure the adequate utilisation of limited resources.

## Simple Summary

Rectal cancer is a relatively common type of cancer. Management of rectal cancer can be complicated by challenges in surgical planning. This can include difficulty identifying candidates likely to have a difficult operation or that might benefit from alternative surgical techniques such as robotic surgery. This research aims to provide a thorough, systematic overview of existing AI tools to support rectal cancer surgical planning by providing preoperative insight into potential surgical difficulty from MRI scans. It identifies 40 studies describing such AI tools, with some demonstrating a good or excellent level of performance. These findings may highlight promising technologies that could advance to more sophisticated clinical trials.

## 1. Introduction

Colorectal cancer (CRC) is the third most common cancer worldwide, representing 10% of all cancer cases, and second for cancer-related deaths [1]. Nearly a third of CRC cases originate in the rectum [2]. Current paradigms in the management of rectal cancer include radiotherapy, chemotherapy (either neoadjuvant or adjuvant), surgery, and, in some cases, immunotherapy [1]. In most cases, surgery for rectal cancer remains the gold standard for potential curative treatment. Over the last three decades, there has been an exponential evolution and innovation in rectal cancer surgery. Open surgery in the pelvis is challenging due to limited views and access, especially in a long, narrow pelvis. The advent of minimally invasive surgery has helped significantly reduce surgical morbidity and postoperative recovery in patients with rectal cancer through better magnified visualisation and the ability to operate in small spaces [3]. Robotic surgery now offers the ability of further increased magnification, with 3D views and instruments that have seven ranges of movement to create fine movements necessary to work within the pelvis. However, robotic surgery is limited to specific centres, and access is an issue due to availability and financial constraints. There is evidence to suggest that robotic rectal cancer resection can produce better outcomes in selected patients [4]. However, identifying those who may benefit based on preoperative surgical planning is difficult to predict.

Prediction of surgical difficulty in rectal cancer depends on several factors, including individual anatomical variations and the rectum’s proximity to critical structures such as pelvic nerves, sphincters and other pelvic organ systems. Tumour-specific factors must also be considered, such as size, specific location within the rectum, extent of invasion into adjacent tissues, and metastatic status (e.g., lymph nodal involvement). Finally, broader patient factors also significantly influence the optimal treatment strategy and complication risks. These may include age, BMI, physiological reserve and co-morbidities, or previous surgical history [5]. A considered understanding of these factors in each case is crucial for both successful outcomes and minimising oncological, surgical, and quality of life complications (e.g., positive resection margins, anastomotic leaks, low anterior resection syndrome). Even when accounting for these factors, morbidity associated with rectal cancer surgery remains high [6]. The nuanced nature of these factors and their impact on patient outcomes highlights the need and significant potential utility of predictive tools to support informed surgical decision-making. To this end, artificial intelligence (AI) tools are being increasingly leveraged across all medical domains to enhance diagnostics, personalise care, and improve our ability to forecast patient outcomes [7]. In conjunction with conventional computing, newer techniques such as deep learning (including neural networks of various architectures) provide opportunities to harness complex medical data, ranging from electronic health records, biochemical results, and radiological images to identify patterns which may be inaccessible to the human clinician. In this context, AI’s potential to analyse pre-operative imaging can enhance decision-making capabilities, guiding surgical teams in the planning or selection of appropriate and case-specific approaches.

Existing AI applied to MRI for pre-operative planning has shown promise across various applications. Segmentation of key anatomical structures and tumours can elucidate the surgical environment; several studies have demonstrated the utility of AI to accurately stage rectal cancer based on preoperative imaging [8] or identify proxy measures of surgical difficulty such as tumour size and location. The incorporation of additional patient data may provide a more accessible and nuanced understanding of potential surgical difficulty for the identification of candidates who stand to benefit the most from specific surgical approaches, such as robotic-assisted techniques, which are designed to support precision in challenging anatomical environments. The incorporation of additional data, such as clinical, biochemical, and derived radiological data, may represent a potential route to gold-standard tools, for which large multimodal AI models may be required. There is growing interest in the use of AI applications for rectal cancer surgery. However, there is a paucity of comprehensive analysis in the form of systematic reviews to synthesise the existing literature into insightful conclusions to guide academic direction. This review therefore aims to provide the first systematic appraisal and synthesis of the current evidence on AI tools applied to MRI for preoperative predictions of surgical difficulty in rectal cancer surgery. We aim to evaluate AI performance metrics, supporting the identification of promising tools which warrant further validation, highlighting evidence gaps in the literature or inadequate adherence to reporting standards, and assessing the readiness of existing tools for integration into clinical workflows, particularly regarding their feasibility and potential benefits for the advancement of surgical decision-making, patient outcomes, and resource utilisation. It will inform our own research going forward to develop a model integrating the most advantageous approaches.

## 2. Methods

A systematic review was undertaken and reported in accordance with the Preferred Reporting Items for Systematic Reviews and Meta-Analyses (PRISMA) guidelines [9] (Appendix A) and was registered in the International Prospective Register of Systematic Reviews (PROSPERO; ID:CRD42024587355).

### 2.1. Searches

Database searches were undertaken on 2nd September 2024 across the following biomedical databases: Medline, Embase, and CENTRAL Trials. A search strategy incorporating three core topics (AI, rectal cancer surgery, and MRI) was designed to account for the broad heterogeneity in the description of AI applications and methodologies. Additionally, a hand search and assessment of the grey literature was planned to ensure all potentially relevant records were returned. The full search strategy is outlined in Appendix A.1.

### 2.2. Study Selection

Records were screened by two independent reviewers at the title and abstract screening stage. Mutually included studies proceeded to a two-reviewer full-text review stage. All conflicts at each stage were resolved through a consensus judgement with the involvement of an independent third reviewer from the study group, an expert in colorectal surgery. Studies were assessed against the inclusion criteria to ensure they (i) concerned adult patient populations, (ii) included patients undergoing rectal cancer surgeries; including trans-anal endoscopic microsurgery (TEMS), trans-anal minimally invasive surgery (TAMIS), total mesorectal excision (TME), anterior resection, abdominoperineal resection, or pelvic exenteration, with all approaches, including open, laparoscopic, and robotic, considered; (iii) related to the development or evaluation of an AI model; (iv) incorporating pre-operative MRI data (allowing any MRI sequence); (v) for the purpose of stratifying surgical difficulty, both directly (difficulty grading predictive models) and indirectly (such as automatic subclassification of the T stage, nodal involvement, and tumour size). Studies were to be excluded if they (i) used non-clinical data sources (e.g., animal or cadaveric studies); (ii) were published prior to 2012 (as a result of the advent of deep learning AI in this year and associated development in model sophistication and capability) [10]; or (iii) were published in a non-English language. The research question, defined as per the PICOTS framework, is provided in Appendix A.2.

### 2.3. Data Collection

Data from the studies identified for inclusion through the full-text review were extracted into a pre-designed database. Extracted data included (i) study characteristics (year of publication, country of origin, study design, participants, comparator, ethical approval, regulatory and public involvement information, funding); (ii) AI model and development details (model type, AI objective, dataset information, training and validation specifics, inputs and outputs); and (iii) outcome measures (including but not limited to accuracy, area under the receiver operating curve, sensitivity, specificity, runtime, and error). Author-reported limitations, including those relating to AI models and included studies themselves, were extracted in full. For quality assurance of this process, data extraction was also undertaken by independent reviewers, with 20% of extractions reviewed by an arbitrator.

### 2.4. Risk of Bias Assessments

The following frameworks were identified for quality assessments, where applicable, to studies identified: the Checklist for Artificial Intelligence in Medical Imaging (CLAIM) [11], the Quality Assessment of Diagnostic Accuracy Studies 2 Tool (QUADAS-2) [12], and the Consolidated Standards of Reporting Trials—Artificial Intelligence (CONSORT-AI) [13].

### 2.5. Synthesis Methods

In anticipation of significant heterogeneity in the identified studies, a narrative synthesis was designed in keeping with Synthesis Without Meta-Analysis (SWiM) reporting guidelines [14] (Appendix B).

Studies were grouped primarily according to AI output (i.e., models providing a difficulty grading, T stage subclassification, and nodal involvement) and secondarily by AI technique (e.g., deep-learning models versus conventional AI) to allow for the best direct comparison. Where provided, standardised outcome metrics across studies (e.g., AUC, sensitivity, specificity) were compared and analysed directly. Data synthesis was primarily descriptive, describing the reporting metrics and examining the key reported findings of each study and any limitations, both identified and author-reported. The synthesis incorporated assessments of study quality, including where concerns in study design or execution limit our ability to draw conclusions from their findings. The summary of key findings was presented in tabular format.

## 3. Results

### 3.1. Study Selection

**Figure 1:**
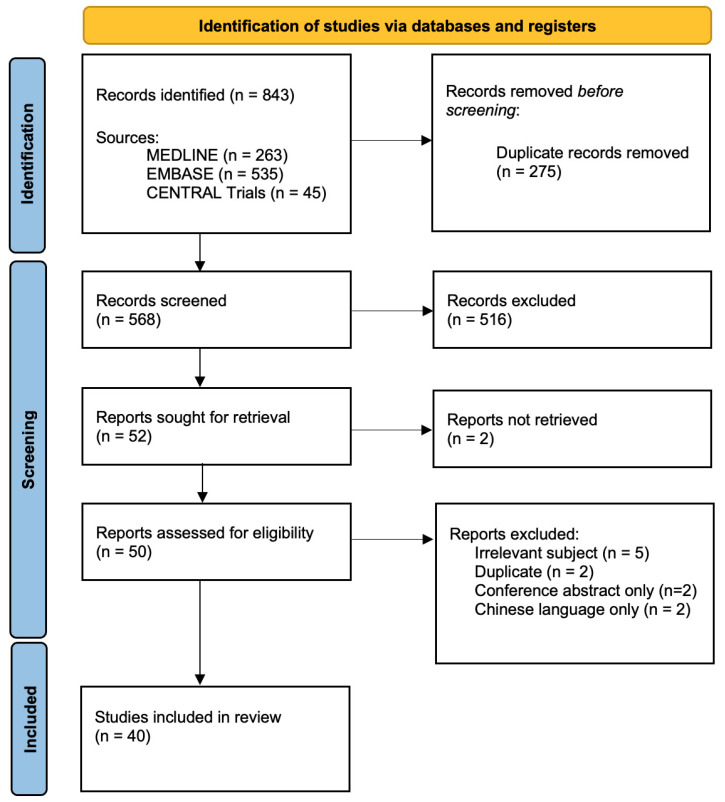
PRISMA flow diagram.

Database searches returned a total of N = 843 records (Medline = 263, Embase = 535, CENTRAL Trials = 45). After duplicates (n = 275) were removed, N = 568 unique studies proceeded to the title and abstract screening, of which N = 50 were mutually included, with a further N = 2 selected following arbitration. Following initial piloting and training, reviewer interrater agreement, quantified in terms of Cohen’s kappa, was 0.748 (95% CI: 0.646–0.836; “substantial agreement”). N = 52 studies were sought for a full-text review, with N = 50 retrieved and N = 40 selected for final inclusion [15,16,17,18,19,20,21,22,23,24,25,26,27,28,29,30,31,32,33,34,35,36,37,38,39,40,41,42,43,44,45,46,47,48,49,50,51,52,53,54] (Figure 1).

### 3.2. Study Characteristics

An overview of the included studies’ characteristics is presented in Table 1. Our review identified a growing rate of studies undertaken in this research area, as expected with the increasing sophistication of AI capabilities. The modal year of publication was 2024, with over a third (n = 14) of studies despite our review’s search execution occurring in September 2024. A majority of the included studies originated from China (n = 35, 88%), with the remainder from Japan (n = 4) and the Netherlands (n = 1). No randomised controlled trials (RCTs) were identified, with most studies employing a single-centre (N = 31, 78%) retrospective study design (N = 35, 88%). Only four studies utilised a prospective design (n = 3 prospective only, with one study undertaking a retrospective model development and testing followed by a prospective external validation of the final model, Fu et al., 2023 [22]).

### 3.3. Overview of the Identified Studies

Our review identified eight distinct categories of AI model objectives (Table 2). The first group, two studies, provided output in the form of a direct surgical difficulty grading, and the remaining seven gave indirect or proxy measures of estimating surgical difficulty pre-operatively. The proxy outputs are predictions of lymph node metastasis/lateral LN metastasis (LNM, N = 17), T staging (N = 12), extramural vascular invasion (EMVI, N = 4), lymphovascular invasion (LVI, N = 2), extranodal extension (ENE, N = 1, defined as breakthrough growth of tumour cells within the LN capsule into surrounding perinodal adipose), perineural invasion (PNI, N = 1), and requirement for multiple linear stapler firings (N = 1). An overview of the design and applications of the studies’ models is provided in Table 2; this includes the model type and whether an image-only approach or clinical data inputs were included. Their full performance data are presented in Table 3. In each category, at least one model was identified with very good performance demonstrated by AUC scores of >0.80 (excluding ENE prediction, AUC = 0.723), with several showing excellent performance considerably greater than this threshold. Studies were typically undertaken with pathologically diagnosed cases selected, and expert radiologists undertook the segmentation or preprocessing of images for AI training where relevant. AUC scores were typically calculated with reference to a model’s accuracy against pathologically confirmed diagnoses, or, where appropriate, radiologists delineated segmentations. A limited number of studies compared model diagnoses against radiologist performance in cases with positive post-surgical specimen pathology. Studies’ comparators and their models’ ground truth inputs and reference standard are provided in Table 2. Judgements on the transparency, risk of bias, and appropriateness of these selections are included in risk of bias assessments shown in Table 4 and Table 5.

#### 3.3.1. Difficulty Grading

The direct difficulty grading studies both used models with different computing architectures, with Yu et al. achieving marginally higher performance with their extreme gradient boosting (“XGBoost”) model design [53]. They used a modified scoring tool proposed by Escal et al. [55] and the Clavien–Dindo classification [56] to provide a value of surgical difficulty. The highest AUC demonstrated in their study was 0.855, with a corresponding sensitivity and specificity of 53.8% and 92.0%, respectively. An added advantage of this model is that it provides a reasonably interpretable output, demonstrating in graphic format to the user which features rank most highly in the determination of surgical difficulty for a given case. By comparison, the model described by Sun et al. [41] achieved an AUC of 0.78.

#### 3.3.2. LN Metastasis

Prediction of LN metastasis was the largest application group. This category was deemed an appropriate proxy for surgical difficulty, as positive status necessitates a more meticulous dissection and can significantly increase surgical challenge and duration due to the fixed pelvic space and critical adjacent structures. Several high-performance models were identified, most notably Li Jin et al. [29] (lateral LN prediction, AUC = 0.994). However, numerous models were identified, achieving excellent performance metrics; this includes Lu et al. 2018 [35] (metastatic LN prediction, AUC = 0.912), Wei et al. (0.929) [47], and Ding et al. [16] and Fang et al. [19] (both 0.912).

#### 3.3.3. T Staging

The second largest grouping was AI for T staging. In our review, a majority of these studies focused on distinguishing between T1–2 and T3–4 tumours to make the key distinction between whether invasion extends beyond the bowel wall into adjacent structures. Models in this domain were very strong, with several providing excellent performance in terms of AUC, sensitivity, and specificity. Fan et al. [18] achieved an AUC of 0.920 (95% CI: 0.829–1.000) with an SVM approach incorporating clinical features, which outperformed the deep learning approach they co-developed alongside this. The single highest performing study in this category was proposed by Wu et al. [50] and demonstrated an AUC of 0.99 when input images were in the horizontal plane, using a deep-learning model capable of sub-classification across all four T stages.

#### 3.3.4. EMVI

The best performer in this category was described by Fang et al. [20], whose AUC was 0.877 in their test set, with a corresponding sensitivity of 100% and a specificity of 84.6%, therefore permitting a high level of confidence in negative predictions made by the model to rule out EMVI. Another high-performance model was provided by Lin et al. [32] with a radiomics-based architecture achieving AUC = 0.865 (95% confidence interval 0.770–0.959), and sensitivity, specificity, and accuracy all greater than 80% (Table 3). The model’s NPV outweighed its PPV (0.897 vs. 0.710), making it a useful tool to segregate cases with a very low probability that EMVI is present to allow surgeons to proceed with confidence in this regard.

#### 3.3.5. LVI

N = 2 studies explored lymphovascular invasion. Both studies employed a radiomics approach, with Wong et al.’s [49] more recent model substantially outperforming that proposed by Fu et al. [21], with AUC = 0.92 (sensitivity 81.2%, specificity 90.0%).

#### 3.3.6. Remaining Application Groups

The remaining categories each contained only one included study: ENE [31], PNI [45], and the prediction of multiple linear stapler firings [22]. The model for PNI prediction [45] was strong (AUC = 0.87); however, the model for ENE [31] prediction requires further development and validation to demonstrate a more reliable, clinically useful performance (AUC: 0.723) owing to its poor sensitivity (54.8%). The final, somewhat more abstracted surrogate marker or operative difficulty is multiple linear stapler firings. The model was evaluated with a robust methodology, including a retrospective testing set followed by an additional prospective recruitment and validation round. The study demonstrated strong performance, most notably an excellent specificity (98.3% in the testing set, 97.3% in the validation cohort). An AUC of 0.88 was achieved in the test set and was well conserved in the prospective validation cohort, where AUC was 0.84. The model’s classification accuracy of 94% was maintained across both testing phases.

### 3.4. Datasets

#### 3.4.1. Training Datasets

A training dataset refers to the dataset used to create a representative model. It is a dataset with known attributes (i.e., case vs. control status) that is used as input to train a model to distinguish and classify later unseen cases [57]. A description of training methods is required by all medical AI reporting standards. The training dataset for each AI model was clearly described in all but three of the included studies [16,24,33]. Of the N = 34 studies describing their training process in terms of patients (“per-patient”), the median number of patients in the training dataset was 150 (interquartile range, IQR = 97–252). The remaining three studies did not describe training on a per-patient basis. One instead used N = 28,080 images of individual metastatic lymph nodes [35]. Another [29] divided their N = 129 patients into multiple images each, with the resulting 319-image metastatic LN+ and 325-image LN− datasets then divided on a per-image basis with 80%:20% for training and testing, respectively. The final [28] treated each side of the patient uniquely, training with a dataset of 139 “patient sides” without stating how many patients’ cases of LN metastasis contributed. As such, these three studies have been excluded from participant calculations.

#### 3.4.2. Test Datasets

The test datasets (analogous to the study population) of the included studies were relatively small with a median of 68 participants (interquartile range, IQR = 50–143.5). Ma et al., 2023 [38] and Sun et al., 2024 [42] both utilised two distinct testing datasets for validation across different centres; as a result of this, the size of each testing set has been treated independently for these calculations. The same has been carried out by Xia et al. [51] but across the three test cohorts they examined. The median of all mean ages of study participants was 60.5 (IQR = 58.45–64). Averaged across all studies, the proportion of study participants that were males was 65.9%.

### 3.5. Ethics/PPI/Governance

Ethical approval was obtained for N = 39 studies, with a further study (Yu et al., 2024 [53]) making no mention of ethical approval. No studies referred to patient or public involvement. Only one study described regulatory input, oversight, or compliance (Fu et al., 2023 [22]), simply stating, “all methods were performed in accordance with the relevant guidelines and regulations”, with no studies addressing the broader regulatory context of software as a medical device (SaMD). This may be on account of the fact that all identified studies represented developmental or evaluation and validation studies for relatively early-stage tools which have not yet been implemented into standard clinical workflows (Table 1).

### 3.6. Appraisal of Studies

The results for the above studies must be interpreted in the context of their study design and potential risk of biases. Detailed quality assessments of included studies are presented in Table 4 (Checklist for Artificial Intelligence in Medical Imaging, CLAIM) and Table 5 (Quality Assessment Tool for Diagnostic Accuracy Studies 2, QUADAS-2).

Regarding CLAIM scoring, the mean score across all studies was 35.65 (tick or N/A) out of 44. The list items most omitted were #39 (failure analysis of incorrectly classified cases, unaddressed in n = 34 studies), #12 (how missing data were handled, n = 34), #11 (de-identification methods, n = 32), #33 (testing on external data, n = 30), and #34 (clinical trial registration, n = 30). Some of these shortcomings may be procedural; for example, that the anonymisation and use of complete medical records without missing data was thought to be implicit, and some reflect that many studies are in their infancy and as such may not be undertaking registerable clinical trials or external validation. Nonetheless, these absences of clarity represent reporting failures. One study [24] is an external validation of the model developed in their earlier study [23], explaining the many “N/A” domains. The QUADAS domain most frequently rated as high risk of bias was patient selection (n = 8 high risk [19,23,25,27,34,39,45,52]), most frequently for potentially inappropriate exclusions of difficult-to-diagnose cases (namely, mucinous rectal cancers), or insufficient clarity regarding patient recruitment. No RCTs were identified by this review, and, as such, CONSORT-AI was not applied.

## 4. Discussion

This review examined current and emerging AI-based tools for supporting objective preoperative surgical difficulty assessments in the context of rectal cancer intervention, with a view to highlighting promising models warranting further research attention and key shortcomings or knowledge gaps within the evidence base. Eight main paradigms within this research field were identified, with the first being (i) direct surgical difficulty grading, and the remainder providing AI outputs in the form of proxy information, which may support radiological evaluations or surgeons’ decision making when making personalised preoperative difficulty evaluations. Those were categorised as (ii) LNM prediction/lateral LNM prediction, (iii) T stage classifiers, (iv) EMVI prediction, (v) LVI prediction, (vi) ENE prediction, (vii) PNI prediction, and (viii) a predictor of the requirement for multiple linear stapler firings.

A majority of the identified studies were included as they provide outputs in the form of proxy indicators for surgical difficulty. For example, T staging provides valuable information about tumour invasion depth and can be formally provided pathologically or estimated clinically using radiological modalities [58]. A majority of T staging studies provided outputs as T1–2 vs. T3–4, a logical and adequate output for difficulty prediction purposes, as a key determinant of how extensive an intervention may be necessitated, particularly considerations of whether multi-visceral or en bloc resections might be indicated [59], which clearly increases surgical complexity. Separately, extramural vascular invasion and lymphovascular invasion status can independently predict poor prognosis [60] and can imply more challenging operative conditions with increased risk of blood vessel involvement and consequent blood loss or tumoural dissemination [61]. ENE and PNI clearly give an impression of tumour invasion and aggressiveness, and cases which may require wider resections or complicated nerve-sparing techniques [62], where surgical duration may suffer as a consequence to preserve the likelihood of complete tumoural resection. Finally, one study examined the likelihood of multiple linear stapler firings, which can be correlated with anatomical constraints or an awkwardly located tumour [63] and is an independent risk factor for anastomotic leak (AL) [64]. The model performed well under robust testing conditions, which, if sustained, may assist determinations that a surgery will have a straightforward execution of double-stapling technique anastomosis and allay concerns of AL related to this portion of the operation or relieve time and resource demands associated with gathering specialist or alternative equipment. It should be noted that the judgement of model performance is largely on the AUC value against a set of gold-standard diagnosed cases, with the model AUC then compared to that of alternative diagnostic approaches, and defining AUC score cut-offs is arbitrary. That said, scores >0.8 are generally considered good or excellent, suggesting a number of identified models across all domains are highly promising candidates to advance into larger, multi-centre, prospective randomised trials [65].

Surgeons performing high-volume rectal cancer operations are well aware of how difficult it is to achieve a successful functional and oncological outcome. An operation often requires a precise dissection in the surgical field because of the proximity of the urogenital structures, prostate gland, vagina, trigone of the bladder, anal sphincter complex, and pelvic floor muscles. Consequently, there are a multitude of surgical options to try and improve the morbidity associated with this type of surgery. Furthermore, choosing the right surgical option can be influenced by the patients’ age, general condition, job, BMI, tumour location, and stage. However, making this decision is complicated by the rapid advancement of new technologies, limited access to specialised resources, varying availability of cutting-edge tools, and the differing levels of expertise among surgeons and surgical teams. In the case of surgical robots, a 2021 study found only 61 robots were available across all 149 acute UK National Health Service (NHS) institutions, with an 84.2% occupancy of them for solely urological procedures [66], demonstrating the necessity of refined, equitable candidate selection and resource distribution. There is evidence to suggest that specific techniques and tools provide better outcomes, but in selected cases. An AI program to suggest which technique would most benefit the patient would be a valuable addition to the decision-making process. This tool would need to incorporate patient-specific factors such as age, overall health, BMI, tumour location, and stage, along with available surgical options and technologies. This would not only optimise functional and oncological outcomes but also reduce the variability in care associated with differences in surgeon expertise and resource availability. Furthermore, such a program could assist in identifying which advanced technologies and tools—whether robotic surgery, minimally invasive techniques, or enhanced imaging methods—would offer the most benefit for each patient’s unique circumstances. Ultimately, the integration of AI into surgical planning could help standardise care, improve efficient use of finite surgical resources, improve surgical outcomes, and reduce the overall morbidity associated with high-complexity rectal cancer surgeries.

Despite these promising early results, the studies in this review had several limitations, many being ubiquitous. A vast majority (N = 35, 88%) of studies were retrospective in their design and therefore at increased risk of selection biases. Additionally, retrospective designs carry a higher risk of overfitting, whereby an AI model does not generalise well from observed training data to unseen or real-world data [67]. The risk of poor generalisation across the identified studies will be further exacerbated by their relatively small training and study populations, as well as their concentration around a few healthcare systems and geographic regions. With nearly 90% of included studies from China, differences in surgical techniques—such as the routine use of lateral lymph node dissection in some Asian centres compared to its selective application in Europe and North America [68]—may influence the relevance and global applicability of AI models trained and validated primarily in Chinese populations. Moreover, although all but two studies [41,42] transparently declared their imaging acquisition approach (CLAIM item 13), these MRI protocols vary significantly between institutions (e.g., field strength, contrast usage, slice thickness), which can impact the generalisability of AI models. Meta-factors such as these only underscore the necessity of larger, more sophisticated research prior to implementation. Furthermore, all studies failed to meaningfully address regulatory requirements, such as the SaMD requirements of the US Food and Drug Administration (FDA) [69] and the analogous Chinese FDA [70] or EU Medical Devices Regulation (EU MDR 2017/745) [71] and In Vitro Diagnostic Medical Devices Regulation (EU IVDR 2017/746) [72]. This may be due to many studies existing in early development, with approval for deployment undertaken on a case-by-case basis for the purposes of research. However, further research work, especially as trials increase in sophistication and clinical integration, should ensure compliance with such requirements. Until such time, the scope for clinical implementation of these tools at scale remains limited. The same is true for the involvement of patient and public consultation to guide implementation, which would represent best practice and is especially pertinent in AI research, which risks scepticism from the public [73]. Criteria for evaluating AI tools in surgery are not uniformly adopted, and standards for evaluation of models moving from “bench” research settings to the bedside in clinical studies must be robust and widely utilised to ensure safe progression towards mainstream practice. Many studies are isolated in their findings, can often originate from computing research groups without reporting in keeping with typical evidence-based medicine standards, or be opaque with regards to their data sources and model architecture. Consequently, the current preparedness of this potentially fruitful field is fragmented and immature. This is evidenced by the absence of rectal cancer-specific AI from the European Union and FDA registers of approved radiological AI tools [74,75]. Existing tools applicable in rectal cancer radiology often support only one domain, such as image segmentation or quantification tasks such as volumetry. Far from the broad, adaptable expertise of radiologists, AI tools’ current scope for implementation in rectal cancer radiology is limited to a purely complementary role, which may support radiologists speed and efficiency or protect against rare human errors or discrepancies [76]. However, progress in the research base may support some of the tools identified to progress towards clinical implementation.

Future directions for research are therefore that further work must aim to expand on high-performing initial models into prospective, larger, multi-centre trials with close adherence to relevant AI in medical research reporting standards and emerging guidelines such as SPIRIT-AI, CONSORT-AI, or DECIDE-AI [77] and the new FUTURE-AI guidelines [78]. Future model development should consider the incorporation of all direct and proxy factors into a much larger model. Beyond this, incorporating a mosaicked analysis of such pre-operative MRI information, integrating tumour characteristics (stage, location, invasion), patient-specific anatomical factors (pelvic dimensions, nerve proximity), and clinical data such as relevant comorbidities, health record entries, vital signs, and biochemical data may allow a reduction in the failure rate. However, failure analysis of incorrectly classified cases, unaddressed in n = 34 studies, was the most omitted CLAIM reporting item. Consequently, confident determinations of their causes are limited. Understanding the specific scenarios where AI models falter is essential for identifying routes to improvement; efforts to make advancements without this understanding risk being wasteful or misguided. External validations should be undertaken wherever possible to demonstrate the robustness of models. Such trials should endeavour to break the geographic clustering seen in this study and recruit balanced and reflective patient populations to ensure generalisability and unbiased training of AI models. Significant expansion in training data will likely be required for high-performance and widely generalisable tools, which must be cautiously balanced against respect for patient confidentiality and patients’ role as stakeholders in both these technologies and their own data. It is not clear from this study how prior treatments (radiotherapy, chemotherapy), which induce substantial changes in tumour morphology and MRI appearance [79], might confound AI models trained on treatment-naïve imaging data, highlighting training data blind spots. Future studies should investigate the impact of prior treatment on model performance, potentially through stratified analyses or the development of models specifically trained on post-treatment MRI data. It is likely that the best course for medical AI development will incorporate multi-disciplinary collaboration between medical and computing experts to achieve harmony in these challenges. Once methodologically optimised evidence exists for these AIs, research to demonstrate the end effect on patient and healthcare system outcomes should be produced to justify final integration into mainstream clinical workflows.

Finally, due to significant heterogeneity in study design and reporting performance metrics, we acknowledge it was not possible to undertake a pooled statistical meta-analysis of the included studies. This limitation will have been exacerbated by our choice to include studies of both deep learning AI methodology and older conventional AI methods despite the inherent difficulty in direct comparison across model architectures. Variability in reference standards and therefore the point of reference for outcome metrics further limits direct comparative validity.

## 5. Conclusions

AI tools for the purpose of preoperative difficulty assessment in the context of rectal cancer surgery are emerging, with promising preliminary results. A multitude of models were identified; however, a majority represent early-stage developmental studies and assessed domains considered to be proxy measures of surgical difficulty assessment. Many identified models are candidates for controlled clinical evaluation, which should be undertaken using robust methodologies and standardised reporting. If developed safely, such AI models may offer enhanced surgical planning, better patient outcomes, and the more efficient allocation of surgical resources, particularly in resource-limited settings.

## Figures and Tables

**Table 1:** Study characteristics of the identified studies.

Characteristic	Value	Frequency (N = 40)
Year of Publication	2016	1
Year of Publication	2017	0
Year of Publication	2018	1
Year of Publication	2019	3
Year of Publication	2020	0
Year of Publication	2021	8
Year of Publication	2022	3
Year of Publication	2023	10
Year of Publication	2024	14
Country of Origin	China	35
Country of Origin	Japan	4
Country of Origin	Netherlands	1
Study Design	Retrospective	36
Study Design	Prospective	3
Study Design	Mixed	1
Study Design	RCT	0
Ethical Approval	Yes	39
Ethical Approval	No	0
Ethical Approval	Not mentioned	1
PPI	Mentioned	0
PPI	Not mentioned	40
Regulatory consideration	Mentioned	1
Regulatory consideration	Not mentioned	39
Funding	Government/National Organisation	20
Funding	University	5
Funding	Government/National Organisation and University	4
Funding	Government/National Organisation and Charitable	3
Funding	Industry	3
Funding	None/Not mentioned	2
Funding	Charitable	1
Funding	Author funded	1
Funding	Hospital and Charitable	1

**Table 2:** Overview of the AI model’s objectives in the identified studies.

Study ID	Model Type	AI Objective/Output	Ground Truth and Comparator Source
Sun et al., 2023 [41]	Deep learning (modified ResNet 50)	Surgical difficulty classification	Surgeon rated difficulty and resected specimen quality.
Yu et al. [53]	extreme gradient boost (XGBoost) (20 MRI measurements, clinical and biochemical data incorporated)	Surgical difficulty classification	Modified Escal [55] surgical difficulty scoring model and Clavien–Dindo classification [56] (See subtext).
Chunli Li et al. [30]	Radiomics (SVM + multivariable logistic regression)	Metastatic LN prediction	Pathologist confirmed diagnosis.
Ding et al. [16]	Deep learning (R-CNN)	Metastatic LN prediction	Radiologist judgement and pathologist judgement.
Dong et al. [17]	Multivariable logistic analysis via backward stepwise selection (Radiomics)	Metastatic LN prediction	Two radiologists blinded (5 and 10 years of experience).
Fang et al. [19]	Radiomics (SVM)	Metastatic LN prediction	Pathologist confirmed diagnosis.
Hao et al. [25]	Radiomics (logistic regression analysis)	Metastatic LN prediction	Radiologist VOI ground truth with pathologist confirmed diagnosis.
Jin Li et al. [29]	Deep transfer learning (CNN)	Metastatic LN prediction	Radiologist performance. Endoscope biopsy and pathologist diagnosis ground truth.
Liu X et al. [54]	Radiomics (SVM + clinical data)	Metastatic LN prediction	Pathologist confirmed diagnosis ground truth. Two expert radiologists review and ROI delineation.
Lu et al. [35]	Deep learning (Faster R-CNN)	Metastatic LN prediction	Pathologist and radiologist confirmed diagnosis.
Ma et al., 2023 [38]	Nomogram	Metastatic LN prediction	Pathologist diagnosed T and N stage.
Niu 2023 et al. [39]	Five classification algorithms (random forest, Gaussian process, Adaboost, K-nearest neighbour, and multilayer perceptron) were compared	Metastatic LN prediction	Pathologist diagnosed TNM stage, EMVI status consensus between two radiologists diagnoses.
Wei et al., 2023 [47]	Logistic regression	Metastatic LN prediction	Pathologist diagnosed lymph node metastasis, two expert radiologists for tumour segmentation and preprocessing and for diagnostic performance comparator.
Wei + Chen et al., 2024 [46]	Random forest	Metastatic LN prediction	Pathological lymph node metastasis + radiologist tumour segmentation, feature extraction, and diagnostic performance comparator.
Kasai et al. [27]	SVM + incorporation of four clinical features (CEA > 5.1, cN ≥ 1, irregular border, mixed signal intensity)	Lateral LNM prediction	Compared to conventional methods (short-axis diameter of the largest lateral lymph node, as detected on MRI), clinically and radiologically diagnosed preoperative reference standard.
Kasai et al. [28]	Deep learning (Sony “Prediction One”)	Lateral LNM prediction	Pathologist confirmed diagnosis.
Sun et al., 2024 [42]	Radiomics (extra trees model)	Lateral LNM prediction	Pathologically identified lateral LNM.
Xia et al. [51]	Deep learning (ResNet-based WISDOM model)	Metastatic LN prediction (and prediction of staging N0–2)	Postoperative specimen pathological LNM status.
Wan et al. [44]	Deep learning (ResNet 101)	Metastatic LN prediction in T1–2 rectal cancers	Pathologist diagnosed LNM T1–2 rectal cancers + compared against three radiologists’ judgement of mrLN status.
Lu et al. [36]	Radiomics (SVM)	T stage (T1–2 vs. T3–4)	Pathologist diagnosed T stage. Two expert radiologists for ROI and feature extraction.
You et al. [52]	SVM	T stage classification (T1–2 vs. T3–4)	Pathologist diagnosed T stage.
Wu et al. [50]	Deep learning (faster R-CNN)	T stage classification	Pathologist diagnosed T stage and radiologist judgement comparator.
Hou et al. [26]	Deep learning	T stage classification (T1–2 vs. T3–4)	Pathologist confirmed diagnosis. Alternative model + expert radiologist comparator.
Tian et al. [43]	Deep learning (3D CNN)	T stage classification (T1–2 vs. T3–4)	Pathologist diagnosed T1–2 vs. T3–4 status, 3x radiologist judgement.
Wei et al., 2024 [48]	Deep learning (multiparametric CNN)	T stage classification (T1–2 vs. T3–4)	Pathologist diagnosed T stage.
Liu et al. [33]	Radiomics (texture analysis, multivariate logistic regression)	T stage classification (T1–2 vs. T3–4) and N stage prediction (N0 vs. N1–2)	Pathologist confirmed diagnosis. Two blinded radiologists completed ROI segmentation. No study comparator.
Ma et al., 2019 [37]	SVM	T stage classification (T1–2 vs. T3–4) and N stage prediction (N0 vs. N1–2)	Pathologist diagnosed T stage.
Fan et al. [18]	Best = SVM + clinical features; (LR/RF/DTL and SVM studied); DTL used ResNet34	T stage classification (T2 vs. T3)	Pathologist diagnosed T stage.
Hamabe et al. [23]	Deep learning (UNet)	Automated segmentation (including tumour, rectum, and mesorectal area), then T2/3 subclassification	Pathologist confirmed diagnosis with expert surgeon created annotations to MRI regions.
Hamabe et al. [24]	Deep learning (UNet)	Automated segmentation (including tumour, rectum, and mesorectal area), then T2/3 subclassification	Accuracy of T staging compared to six radiologists (depth, LNs, mrEMVI, mrCRM) and pathological ground truth.
Lin et al. [32]	Radiomics (logistic regression + clinical data)	EMVI prediction	Pathologist confirmed diagnosis. Two blinded radiologists completed ROI segmentation. No study comparator.
Liu S et al. [34]	Radiomics (SVM + clinical data)	EMVI prediction	Pathologist confirmed diagnosis ground truth. Two expert radiologists review and ROI delineation.
Shu et al. [40]	Bayes	EMVI prediction	Pathologist confirmed diagnosis.
Cai et al. [15]	Deep learning (CNN)	EMVI prediction (and CR prediction)	Radiologist judgement.
Fang et al. [20]	Multivariable logistic regression	EMVI prediction (and prognosis prediction)	Pathologist confirmed diagnosis.
Fu et al. [21]	Radiomics (ridge classifier for feature extraction, linear SVC for classifier)	LVI prediction	Pathologist confirmed diagnosis.
Wong et al. [49]	Radiomics	LVI prediction	Pathologist confirmed diagnosis.
Li et al. [31]	Radiomics (univariate and multivariate Cox regression analyses were used to construct a prognostic model for assessing 3-year RFS; the diagnostic performance of this prognostic model was determined using time-dependent ROC curves)	Extranodal extension prediction (+ prognosis 3y RFS)	Two radiologist judgements for ENE status and tumour segmentation, pathology diagnosis ground truth.
Wang et al. [45]	SVM	Prediction of PNI	Pathologist confirmed diagnosis.
Fu et al. [22]	Deep learning (mask region-based CNN and binary logistic regression)	Prediction of multiple (≥3) linear stapler firings	Pathologist confirmed diagnosis.

Abbreviations: support vector machine (SVM), convolutional neural network (CNN), logistic regression (LR), random forest (RF), deep transfer learning (DTL), carcinoembryonic antigen (CEA), lymph node (LN), lymph node metastasis (LNM), lymphovascular invasion (LVI), extramural vascular invasion (EMVI), and extranodal extension (ENE). Yu et al. [53]; surgical difficulty score: modified surgical difficulty score as per Escal et al. [55]; surgical difficulty score: duration of surgery > 240 min (3 points), blood loss > 200 mL (1 point), conversion to laparotomy (3 points), postoperative complications (grade II and III) (1 point), use of transanal dissection (2 points), and postoperative hospital stay > 12 days (2 points). Patients were divided into two groups: low surgical difficulty group (<6 points) and high surgical difficulty group (≥ 6 points). The postoperative complications were graded according to the Clavien–Dindo classification [56]: grade II: medical treatment is required, including blood transfusion or total parenteral nutrition. Grade III: surgical, endoscopic, or radiological intervention is required.

**Table 3:** Performance metrics of the AI models in the identified studies.

Study ID and Year	AI Objective	AUC (95% CI)	Sensitivity% (95% CI)	Specificity% (95% CI)	Accuracy%	Other Metrics/Comments
Sun (2023) [41]	Surgical difficulty classification	0.78	Not specified	75	80	F1 = 0.815, Precision 78.6%, Recall 84.6%
Yu (2024)[53]	Surgical difficulty classification	0.855	53.8	92	83	Incorporates seven variables, with an interpretable model output. Tumour height, prognostic nutrition index, pelvic inlet and outlet, sacrococcygeal distance, mesorectal fat area, and angle 5.
Chunli Li (2021) [30]	Metastatic LN prediction	0.884	83.3	82.9	77	
Ding (2019) [16]	Metastatic LN prediction	0.912	Not specified	Not specified	78	
Dong (2023) [17]	Metastatic LN prediction	0.62 (0.49–0.74)	39.6	78.8	62	PPV = 75.9%, NPV 42.6%
Fang (2023) [19]	Metastatic LN prediction	0.912 (0.771–1.000)	90	75	80.8	PPV 69.20%, NPV 92.30%
Hao (2024) [25]	Metastatic LN prediction	0.739	50	78.9	83	
Jin Li (2021) [29]	Metastatic LN prediction	0.994	95.3	95.2	75	PPV 95.2%, NPV 95.3%
Liu X (2021) [54]	Metastatic LN prediction	0.832 (0.717–0.915) [*p* = 0.015]	81.5	75	82	PPV = 0.710, NPV = 0.844
Lu (2018) [35]	Metastatic LN prediction	0.912	Not specified	Not specified	74	-
Ma 2023 (2023) [38]	Metastatic LN prediction	0.908	81.8	94.9	85	PPV = 64.7%, NPV = 86.9%
Niu 2023 (2023) [39]	Metastatic LN prediction	0.785	62.1	73.2	81	
Wei (2023) [47]	Metastatic LN prediction	0.929	90	88	85	
Wei + Chen 2024 (2024) [46]	Metastatic LN prediction	0.83	60	94.3	90	
Kasai (2021) [27]	Lateral LNM prediction	0.903 (0.832–0.974) [*p* < 0.001]	90	79.4	76	
Kasai (2024) [28]	Lateral LNM prediction	0.781	75	66.7	84	PPV 25%, NPV = 94.7%
Sun 2024 (2024) [42]	Lateral LNM prediction	Centre 1: 0.741, Centre 2: 0.713	Centre 1—62%, Centre 2—58%	Centre 1—81%, Centre 2 90%	89	F1 Centre 1: 70%, Centre 2: 71%
Xia (2024) [51]	Metastatic LN prediction (and prediction of staging N0–2)	0.81	70.2	84	84	MAE = 1.049
Wan (2023) [44]	Metastatic LN prediction in T1–2 rectal cancers	0.79	100	66	79	
Hamabe (2022) [23]	Automated segmentation (including tumour, rectum, and mesorectal area), then T2/3 subclassification	N/A	77.3	76.8	88	Mean DSC for tumour/rectum/mesorectum = 0.727/0.930/0.917
Hamabe (2024) [24]	Automated segmentation (including tumour, rectum, and mesorectal area), then T2/3 subclassification	N/A	76.8	76.7	91	Across whole set (Inc. non-Philips scanners and all interslice spacings) Accuracy 68.5%, Sensitivity 70.6%, Specificity 61.3%
Lu (2021) [36]	T stage (T1–2 vs. T3–4)	0.903 (0.807–0.999)	87	82.3	78	PPV = 87%, NPV = 82.3% in best overall model (validation set, method 2)
You (2021) [52]	T stage classification (T1–2 vs. T3–4)	0.91	90	88.57	78	
Wu (2021) [50]	T stage classification	0.99	Not specified	Not specified	82	Compared to a stated 86% accuracy of manual MRI diagnosis
Hou (2023) [26]	T stage classification (T1–2 vs. T3–4)	0.869	71.1	93.1	82	Better than study comparator radiologist performance, *p* < 0.05
Tian (2023) [43]	T stage classification (T1–2 vs. T3–4)	0.958	100	92.9	84	PPV 92.3%, NPV 100%
Wei (2024) [48]	T stage classification (T1–2 vs. T3–4)	0.854	81.8	82.9	88	F1 = 0.82
Liu (2016) [33]	T stage classification (T1–2 vs. T3–4) and N stage prediction (N0 vs. N1–2)	0.751 (0.637–0.865)	96.2	45.3	79	
Ma (2019) [37]	T stage classification (T1–2 vs. T3–4) and N stage prediction (N0 vs. N1–2)	0.862	83.3	85	73	
Fan (2024) [18]	T stage classification (T2 vs. T3)	0.920 (0.829–1.000). Deep transfer learning model = 0.885 (0.764–1.000)	SVM 100%, DTL 98.8%	SVM 82.6, DTL 69.6	92	*p* = 0.035 for DTL data.
Lin (2024) [32]	EMVI prediction	0.865 (0.770–0.959)	81.8	83.3	88	PPV = 0.720, NPV = 0.897
Liu S (2021) [34]	EMVI prediction	0.834	70.8	89.2	80	
Shu 2022 (2022) [40]	EMVI prediction	0.835	71.4	88.5	83	
Cai (2024) [15]	EMVI prediction (and CR prediction)	0.76 (0.66–0.84)	67 (55–76)	70 (59–80)	85	PPV 67% (52–80), NPV 69% (55–80), F1 0.67 (0.54–0.78)
Fang (2022) [20]	EMVI prediction (and prognosis prediction)	0.877	100	84.6	87	
Fu (2019) [21]	LVI prediction	0.74	Not specified	Not specified	81	
Wong (2024) [49]	LVI prediction	0.92	81.2	90	86	
Li (2024) [31]	Extranodal extension prediction (+ prognosis 3y RFS)	0.723	54.8	87.5	86	
Wang (2024) [45]	Prediction of PNI	0.87	76.9	65	87	F1: 66.7%, PPV: 58.8%, NPV: 81.25%
Fu (2023) [22]	Prediction of multiple (≥3) linear stapler firings	0.88 (test set), 0.84 (validation set)	70 (test set), 68.8 (validation cohort)	98.3 (test set), 97.3 (validation cohort)	94.1 (test set), 93.8 (validation cohort)	

Abbreviations: area under the receiver operating curve (AUC), deep transfer learning (DTL), Dice similarity coefficient (DSC), mean absolute error (MAE), positive predictive value (PPV), negative predictive value (NPV), lymph node (LN), lymph node metastasis (LNM), lymphovascular invasion (LVI), extramural vascular invasion (EMVI), and recurrence-free survival (RFS).

**Table 4:** Risk of bias scoring—CLAIM. See here for details: https://pubs.rsna.org/doi/10.1148/ryai.240300 (accessed on 10 November 2024).

Study ID	1	2	3	4	5		6		7		8		9		10			11		12		13		14		15		16		17		18		19	
CLAIM Item	Cai [15]	Ding [16]	Dong [17]	Fan [18]	Fang 22 [20]		Fang 23 [19]		Fu 19 [21]		Fu 23 [22]		Hamabe 22 [23]		Hamabe 24 [24]			Hao [25]		Hou [26]		Kasai 21 [27]		Kasai 24 [28]		Jin Li 21 [29]		Chunli Li 21 [30]		Li [31]		Lin [32]		Liu 16 [33]	
1	✓	✓	✓	✓	✓		✓		✓		✓		✓		✓			✓		✓		✓		✓		✓		✓		✓		✓		✓	
2	✓	✓	✓	✓	✓		✓		✓		✓		✓		✓			✓		✓		✓		✓		✓		✓		✓		✓		✓	
3	✓	✓	✓	✓	✓		✓		✓		✓		✓		✓			✓		✓		✓		✓		✓		✓		✓		✓		✓	
4	✓	✓	✓	✓	✓		✓		✓		✓		✓		✓			✓		✓		✓		✓		✓		✓		✓		✓		✓	
5	✓	✓	✓	✓	✓		✓		✓		✓		✓		✓			✓		✓		✓		✓		✓		✓		✓		✓		✓	
6	✓	✓	✓	✓	✓		✓		✓		✓		✓		✓			✓		✓		✓		✓		✓		✓		✓		✓		✓	
7	✓	✓	✓	✓	✓		✓		✓		✓		✓		✓			✓		✓		✓		✓		✓		✓		✓		✓		✓	
8	✓	✓	✓	✓	✓		✓		✓		✓		✕		✓			✓		✓		✓		✓		✓		✓		✓		✓		✓	
9	✓	✓	✓	✓	✓		✓		✓		✓		✓		✓			✓		✓		✓		✓		✓		✓		✓		✓		✓	
10	✓	✓	✓	✓	✓		✓		✓		✓		✕		N/A			✓		✓		✓		✓		✓		✓		✓		✓		✓	
11	N/A	✕	✓	✕	✕		✕		✕		✕		✓		✕			✕		✕		✓		✕		✕		✕		✕		✕		✕	
12	✕	✓	✕	N/A	✕		✕		✕		✕		✕		✕			✕		✕		✕		✕		✕		✕		✓		✓		✕	
13	✓	✓	✓	✓	✓		✓		✓		✓		✓		✓			✓		✓		✓		✓		✓		✓		✓		✓		✓	
14	✓	✓	✓	✓	✓		✓		✓		✓		✓		✓			✓		✓		✓		✓		✓		✓		✓		✕		✓	
15	✓	N/A	N/A	N/A	N/A		N/A		N/A		N/A		✓		✓			N/A		N/A		✕		✕		N/A		✓		N/A		✕		N/A	
16	✓	✓	✓	✓	✓		✓		✓		✓		✓		✓			✓		✓		✓		✓		✓		✓		✓		✕		✓	
17	✓	✕	✓	✕	✓		✕		✕		✕		✓		✓			✕		✕		✕		✕		✕		✕		✕		✕		✕	
18	N/A	✓	✓	✕	✓		✓		✕		✕		✕		N/A			✓		✓		✕		✕		✓		✓		✓		✕		✓	
19	✓	✕	✓	✓	✓		✓		✓		✓		✓		N/A			✓		✓		✓		✓		✓		✓		✓		✓		✕	
20	✓	✕	✓	✓	✓		✓		✓		✓		✓		N/A			✓		✓		✓		✓		✓		✓		✓		✓		✕	
21	✓	✓	✓	✓	✓		✓		✓		✓		✓		✓			✓		✓		✓		✓		✓		✓		✓		✓		✓	
22	✓	✓	✓	✓	✓		✓		✓		✓		✓		N/A			✓		✓		✓		✓		✓		✓		✕		✓		✓	
23	✓	✓	✓	✓	✕		✓		✕		✓		✓		N/A			✓		✓		✓		✓		✓		✓		✓		✓		✓	
24	✓	N/A	✓	✓	✓		✓		✓		✓		✓		N/A			✓		✓		✓		✓		✓		✓		✓		✓		✓	
25	✓	N/A	✓	✓	✓		✓		✓		✓		✓		N/A			✓		✓		✓		✓		✓		✓		✓		✕		✕	
26	✓	N/A	✓	✓	✓		✓		✓		✓		✓		N/A			✓		✓		✕		✓		N/A		✓		✓		✓		✓	
27	✓	N/A	✓	✓	✓		✓		✓		✓		✓		N/A			✓		✓		✓		✓		✓		✓		✓		✓		✓	
28	✓	✓	✓	✓	✓		✓		✓		✓		✓		✓			✓		✓		✓		✓		✓		✓		✓		✓		✓	
29	✓	✓	✓	✓	✓		✓		✕		✓		✓		✓			✓		✓		✓		✕		✓		✓		✓		✓		✓	
30	✕	✕	✕	✕	✕		✕		✕		✕		✕		✓			✕		✕		✕		✕		✕		✕		✕		✕		✕	
31	✓	✕	✕	✓	✕		✕		✓		N/A		N/A		N/A			✕		✕		✓		✕		N/A		✕		✕		✓		N/A	
32	✓	N/A	✓	✓	✓		✓		✓		✓		✓		N/A			✓		✓		✓		✓		✓		✓		✓		✓		✓	
33	✓	✓	✕	✕	✕		✕		✕		✓		✕		✓			✕		✕		✕		✓		✕		✕		✕		✕		✕	
34	N/A	✓	✕	✕	✕		✕		✕		✕		✕		✓			✕		✕		✓		✕		✕		✕		✕		✓		✕	
35	✓	✓	✓	✓	✓		✓		✓		✓		✓		✓			✓		✓		✓		✓		✓		✓		✓		✓		✓	
36	✓	✓	✓	✓	✓		✓		✓		✓		✓		✓			✓		✓		✓		✓		✓		✓		✓		✓		✓	
37	✓	✓	✓	✓	✓		✓		✕		✓		✓		✓			✓		✓		✓		✕		✓		✓		✓		✓		✓	
38	✓	✓	✓	✓	✓		✓		✓		✓		✓		✓			✓		✓		✓		✓		✓		✓		✓		✓		✓	
39	✓	✕	✕	✕	✕		✕		✕		✕		✕		✕			✕		✕		✕		✕		✕		✕		✕		✕		✕	
40	✓	✕	✓	✓	✓		✓		✓		✓		✓		✓			✓		✓		✓		✓		✓		✓		✓		✓		✓	
41	✓	✓	✓	✓	✓		✓		✓		✓		✓		✓			✓		✓		✓		✓		✓		✓		✓		✓		✓	
42	✓	✓	✕	✕	✕		✕		✕		✕		✕		✓			✕		✕		✓		✓		✕		✕		✕		✕		✕	
43	✓	✓	✓	✓	✓		✕		✕		✓		✓		✕			✓		✓		✓		✓		✕		✓		✓		✓		✕	
44	✓	✓	✓	✓	✓		✓		✓		✓		✓		✓			✓		✓		✕		✓		✓		✓		✓		✓		✓	
TOTAL/44	39	30	36	34	34		33		30		34		34		28			34		34		35		33		32		35		34		33		30	
Number N/A	3	6	1	2	1		1		1		2		1		12			1		1		0		0		3		0		1		0		2	
Adjusted Total	42	36	37	36	35		34		31		36		35		40			35		35		35		33		35		35		35		33		32	
	**20**	**21**	**22**	**23**	**24**	**25**		**26**		**27**		**28**		**29**		**30**	**31**		**32**		**33**		**34**		**35**		**36**		**37**		**38**		**39**		**40**
CLAIM Item	Liu X 21 [54]	Liu S 21 [34]	Lu 18 [35]	Lu 21 [36]	Ma 19 [37]	Ma 23 [38]		Niu 2023 [39]		Shu 2022 [40]		Sun 23 [41]		Sun 24 [42]		Tian [43]	Wan [44]		Wang 24 [45]		Wei and Chen 2024 [46]		Wei 23 [47]		Wei (Deep) 24 [48]		Wong [49]		Wu [50]		Xia [51]		You [52]		Yu [53]
1	✓	✓	✓	✓	✓	✓		✓		✓		✓		✓		✓	✓		✓		✓		✓		✓		✓		✓		✓		✓		✓
2	✓	✓	✓	✓	✓	✓		✓		✓		✓		✓		✓	✓		✓		✓		✓		✓		✓		✓		✓		✓		✓
3	✓	✓	✓	✓	✓	✓		✓		✓		✓		✓		✓	✓		✓		✓		✓		✓		✓		✓		✓		✓		✓
4	✓	✓	✓	✓	✓	✓		✓		✓		✓		✓		✓	✓		✓		✓		✓		✓		✓		✓		✓		✓		✓
5	✓	✓	✕	✓	✓	✓		✓		✓		✓		✓		✓	✓		✓		✓		✓		✓		✓		✓		✓		✓		✓
6	✓	✓	✓	✓	✓	✓		✓		✓		✓		✓		✓	✓		✓		✓		✓		✓		✓		✓		✓		✓		✓
7	✓	✓	✓	✓	✓	✓		✓		✓		✓		✓		✓	✓		✓		✓		✓		✓		✓		✓		✓		✓		✓
8	✓	✓	✕	✓	✓	✓		✓		✓		✓		✓		✓	✓		✓		✓		✓		✓		✓		✓		✓		✕		✓
9	✓	✓	✓	✓	✓	✓		✓		✓		✓		✓		✓	✓		✓		✓		✓		✓		✓		✓		✓		✕		✓
10	✓	✓	✓	✓	✓	✓		✓		✓		✓		✓		✓	✓		✓		✓		✓		✓		✓		✓		✓		✓		✓
11	✕	✕	✓	✕	✕	✕		✕		✕		✕		✕		✕	✓		✕		✓		✕		✕		✕		✓		✕		✕		✕
12	✕	✓	✕	✕	✕	✕		✕		✓		✕		✕		✕	✕		✕		✕		✕		✕		✕		✕		✕		✕		✕
13	✓	✓	✓	✓	✓	✓		✓		✓		✕		✕		✓	✓		✓		✓		✓		✓		✓		✓		✓		✓		✓
14	✓	✓	✓	✓	✓	✓		✓		✓		✓		✓		✓	✓		✓		✓		✓		✓		✓		✓		✓		✓		✓
15	N/A	N/A	✓	N/A	N/A	N/A		N/A		N/A		✓		N/A		N/A	N/A		✓		✓		N/A		✓		✓		✓		✓		N/A		N/A
16	✓	✓	✓	✓	✓	✓		✓		✓		✓		✓		✓	✓		✓		✓		✓		✓		✓		✓		✓		✓		✓
17	✕	✕	✓	✓	✓	✓		✓		✓		✓		✓		✓	✓		✓		✓		✓		✓		✓		✓		✓		✓		✓
18	✓	✕	✕	✓	N/A	✓		✕		✓		✕		N/A		✓	✕		✕		✓		✕		✓		✕		✓		✕		✓		✕
19	✓	✓	✓	✓	✓	✓		✓		✓		✓		✓		✓	✓		✓		✓		✓		✓		✓		✓		✓		✕		✓
20	✓	✓	✓	✓	✓	✓		✓		✓		✓		✓		✓	✓		✓		✓		✓		✓		✓		✕		✓		✓		✓
21	✓	✓	✓	✓	✓	✓		✓		✓		✓		✓		✓	✓		✓		✓		✓		✓		✓		✓		✓		✓		✓
22	✓	✓	✓	✓	✓	✓		✓		✓		✓		✓		✓	✓		✓		✓		✓		✓		✓		✓		✓		✓		✓
23	✓	✓	✓	✓	✓	✓		✕		✓		✓		✓		✕	✓		✓		✓		✓		✕		✓		✓		✓		✓		✓
24	✓	✓	✓	✓	✓	✓		✓		✓		✓		✓		✓	✕		✓		✓		✓		✓		✓		✓		✓		✓		✓
25	✓	✓	✓	✓	✓	✓		✓		✓		✓		✓		✓	✓		✓		N/A		✓		✓		✓		✓		✓		✕		✓
26	✓	✓	N/A	✓	✓	N/A		✓		✓		✓		✓		✓	✓		✓		✓		✓		✓		✓		N/A		✓		✓		✓
27	✓	✓	N/A	✓	✓	N/A		✓		✓		✓		✓		✓	N/A		✓		✓		✓		✕		✕		N/A		✓		✓		N/A
28	✓	✓	✓	✓	✓	✓		✓		✓		✓		✓		✓	✓		✓		✓		✓		✓		✓		✓		✓		✓		✓
29	✓	✓	✕	✓	✓	✓		✓		✓		✓		✓		✓	✓		✓		✓		✓		✓		✓		✕		✓		✓		✓
30	✕	✕	✕	✕	✕	✕		✕		✕		✕		✕		✓	✕		✕		✓		✓		✓		✓		✕		✕		✕		✕
31	✓	✕	✕	✕	✕	✕		✕		✕		✓		✓		✕	✕		✕		✓		✓		✕		✕		✕		✓		N/A		✕
32	✓	✓	✓	✓	✓	✓		✓		✓		✓		✓		✓	✓		✓		✓		✓		✓		✓		✓		✓		✓		✓
33	✕	✕	✕	✕	✕	✓		✕		✓		✕		✕		✕	✕		✕		✓		✕		✕		✕		✕		✓		✓		✕
34	✕	✕	✓	✕	✕	✓		✕		✕		✕		✕		✕	✕		✕		✕		✓		✕		✕		✓		✕		✓		✕
35	✓	✓	✕	✓	✓	✓		✕		✓		✕		✕		✓	✓		✕		✓		✓		✓		✕		✕		✓		✓		✓
36	✓	✓	✕	✓	✓	✓		✓		✓		✓		✓		✓	✓		✓		✓		✓		✓		✓		✕		✓		✓		✓
37	✓	✓	✓	✓	✓	✓		✓		✓		✓		✓		✓	✓		✓		✓		✓		✓		✓		✓		✓		✓		✓
38	✓	✓	✕	✓	✓	✓		✓		✓		✓		✓		✓	✓		✓		✓		✓		✓		✓		✕		✓		✓		✕
39	✕	✕	✕	✕	✕	✕		✕		✕		✓		✓		✓	✕		✕		✕		✕		✓		✕		✕		✕		✓		✕
40	✓	✓	✓	✓	✓	✓		✓		✓		✓		✓		✓	✓		✓		✓		✓		✓		✓		✓		✓		✕		✓
41	✓	✓	✓	✓	✓	✓		✓		✓		✓		✓		✓	✓		✓		✓		✓		✓		✓		✓		✓		✓		✓
42	✕	✕	✕	✓	✕	✓		✓		✓		✓		✓		✓	✓		✓		✓		✕		✓		✓		✕		✓		✓		✕
43	✓	✓	✓	✓	✕	✓		✓		✕		✓		✓		✓	✓		✕		✓		✕		✕		✓		✓		✓		✓		✓
44	✓	✓	✓	✓	✓	✓		✓		✓		✓		✓		✓	✓		✓		✓		✓		✓		✓		✓		✓		✓		✓
TOTAL/44	35	34	29	36	33	36		33		37		36		35		37	34		34		40		36		36		35		31		38		34		32
Number N/A	1	1	2	1	2	3		1		1		0		2		1	2		0		1		1		0		0		2		0		2		2
Adjusted Total	36	35	31	37	35	39		34		38		36		37		38	36		34		41		37		36		35		33		38		36		34

**Table 5:** Risk of bias score—QUADAS-2. See here for details: https://www.bristol.ac.uk/population-health-sciences/projects/quadas/quadas-2/ (accessed on 10 November 2024).

Study ID	Year	Patients	Index Test	Reference Standard	Flow and Timing	PART B: Patients	Part B: Index	Part B: Reference	Comments
Cai [15]	2024	Low risk	Low risk	**High risk**	Low risk	Low risk	Low risk	**High risk**	Ref standard—uses biopsy-proven rectal Ca (low risk); however, determines EMVI status by radiology, not biopsy (as patients who underwent CRT would not be representative of their baseline if using biopsy).
Ding [16]	2019	Low risk	Low risk	Low risk	Low risk	Low risk	Low risk	Low risk	*
Dong [17]	2023	Low risk	Low risk	Low risk	Low risk	Low risk	Low risk	Low risk	*
Fan [18]	2024	Low risk	Low risk	Low risk	Low risk	Low risk	Low risk	Low risk	*
Fang [20]	2022	Low risk	Low risk	Low risk	Low risk	Low risk	Low risk	Low risk	*
Fang [19]	2023	**High risk**	Low risk	Low risk	Low risk	Low risk	Low risk	Low risk	Exclusion of mucinous cases.
Fu [21]	2019	Low risk	Low risk	Low risk	Low risk	Low risk	Low risk	Low risk	*
Fu [22]	2023	Low risk	Low risk	Low risk	Low risk	Low risk	Low risk	Low risk	*
Hamabe [23]	2022	**High risk**	Low risk	Low risk	**Unclear risk**	Low risk	Low risk	Low risk	Patients: High, only due to omission of clear inclusion/exclusion criteria. Unclear on the timeframe between recruitment, surgery, pathology, and MRI image acquisition in general or on a per-patient basis.
Hamabe [24]	2024	Low risk	Low risk	Low risk	**Unclear risk**	Low risk	**High risk**	Low risk	Unclear on the timeframe between recruitment, surgery, pathology, and MRI image acquisition in general or on a per-patient basis. N = 4 cases from a separate institution had images acquired with a different MRI scanner despite being analysed together, which may lead to variation in performance.
Hao [25]	2024	**High risk**	Low risk	Low risk	Low risk	Low risk	**Unclear risk**	Low risk	Excluded patients with non-adenocarcinoma and mucinous tumours may contribute to over-optimistic performance data. Variations in the MRI scanners used across the patient set may lead to applicability risk re: index test; however, this was acknowledged and sub-grouped for analysis.
Hou [26]	2023	Low risk	Low risk	Low risk	Low risk	**High risk**	Low risk	Low risk	Very small tumours ("too tiny for segmentation") were excluded, which may lead to more optimistic performance measures.
Kasai [27]	2021	**High risk**	Low risk	**High risk**	**Unclear risk**	Low risk	Low risk	**High risk**	Selection bias—training vs. test set had some significant differences observed across several learning items. Radiologic diagnosis was the predominant reference data rather than pathologically confirmed—authors describe this as a comparison to the “conventional method of using LLN diameter”. Unclear timeframe between preoperative MRI and clinical data used and corresponding operative/postoperative classification, data, and images.
Kasai [28]	2024	Low risk	**High risk**	Low risk	**Unclear risk**	Low risk	Low risk	Low risk	Pathologically determined status (low risk ref. std.); however, incorporation of EMVI data during index test taken by radiologist and surgeon judgement is not biopsy proven. Unclear timeframe between preoperative MRI and remainder of data.
Jin Li [29]	2021	Low risk	Low risk	Low risk	Low risk	Low risk	Low risk	Low risk	
Chunli Li [30]	2021	Low risk	Low risk	Low risk	**Unclear risk**	Low risk	Low risk	Low risk	Unclear timeframe between MRI acquisition and remainder of patient flow.
Li [31]	2024	Low risk	Low risk	Low risk	Low risk	Low risk	Low risk	Low risk	
Lin [32]	2024	Low risk	Low risk	Low risk	Low risk	Low risk	Low risk	**Unclear risk**	Pathologically confirmed rectal Ca, unclear if pathologically determined EMVI status as reference (implied to be pathologically determined but not explicitly stated).
Liu [33]	2016	**Unclear risk**	Low risk	Low risk	**Unclear risk**	Low risk	Low risk	Low risk	Patients—potentially inappropriate exclusions, e.g., endoscopic resections, palliative resections. One-month window (rather than the more common two weeks) as cut-off for time between MRI and resection = unclear, potentially increased the risk of bias in the flow and timing domains.
Liu X [54]	2021	Low risk	Low risk	Low risk	Low risk	Low risk	Low risk	Low risk	
Liu S [34]	2021	**High risk**	Low risk	Low risk	Low risk	Low risk	Low risk	Low risk	Exclusion of patients with mucinous tumours may bias results to over-optimistic performance (much more hyperintense signal with mucinous tumours, therefore may require unique models and be a necessary exclusion); this is countered by a robust, balanced, and consecutive recruitment of patients from a prospectively assembled database.
Lu [35]	2018	**Unclear risk**	Low risk	High risk	Low risk	Unclear risk	Low risk	**Unclear risk**	The only inclusion was patients with rectal cancer and LNM on MRI; no other data recorded; reference standard was radiological LNM by two radiologists and one pelvic surgeon = no interoperator agreement recorded.
Lu [36]	2021	Low risk	Low risk	Low risk	Low risk	Low risk	Low risk	Low risk	
Ma 2023 [38]	2023	Low risk	Low risk	Low risk	Low risk	Low risk	Low risk	Low risk	
Ma 2019 [37]	2019	Low risk	Low risk	Low risk	Low risk	Low risk	Low risk	Low risk	
Niu 2023 [39]	2023	**High risk**	Low risk	Low risk	Low risk	Low risk	Low risk	**High risk**	Excluded mucinous RC, EMVI radiologically identified (was a model input vs. study outcome).
Shu 2022 [40]	2022	Low risk	Low risk	Low risk	**Unclear risk**	Low risk	Low risk	Low risk	
Sun 2024 [42]	2024	Low risk	**Unclear risk**	Low risk	**Unclear risk**	**High risk**	Low risk	Low risk	Inclusion criteria was RC patients whose MRI documented LLNs exceeded 5 mm in short-axis diameter—bias risk due to exclusion of smaller lymph nodes vs. using pathological N stage to decide inclusion, unclear if radiologist delineating ROIs blinded, unclear if EMVI was radiographic or pathological. Unclear timeframe.
Sun [41]	2023	Low risk	**Unclear risk**	**High risk**	**Unclear risk**	Low risk	Low risk	Low risk	Subjective surgical difficulty is decided based on pelvic anatomy as per the operating surgeon based on grades 1–4—risk of bias vs. more quantitative measures—surgery time, blood loss, etc. Unclear timeframe from MRI to surgery. Unclear if ROI annotators are blinded to results.
Tian [43]	2023	Low risk	Low risk	Low risk	Low risk	Low risk	Low risk	Low risk	
Wang [45]	2024	**High risk**	Low risk	Low risk	Low risk	Low risk	Low risk	Low risk	Excluded mucinous rectal cancers.
Wan [44]	2023	Low risk	Low risk	Low risk	Low risk	Low risk	**Unclear risk**	Low risk	Total of 10 mg intramuscular raceanisodamine hydrochloride (reduces muscle spasm) use prior to imaging, except if contraindications exist—not specified how many cases this included.
Wei + Chen 2024 [46]	2024	Low risk	Low risk	Low risk	Low risk	Low risk	Low risk	Low risk	
Wei [47]	2023	Low risk	**High risk**	Low risk	Low risk	Low risk	Low risk	Low risk	Radiologic EMVI—not an output but was a model input for prediction LNM.
Wei (deep) [48]	2024	Low risk	Low risk	Low risk	**Unclear risk**	Low risk	Low risk	**Unclear risk**	Unclear timeframe between MRI and surgery, EVMI was used in the model but it is unclear whether this was pathological EMVI vs. radiological.
Wong [49]	2024	Low risk	Low risk	Low risk	Low risk	Low risk	Low risk	Low risk	
Wu [50]	2021	Low risk	Low risk	Low risk	**Unclear risk**	Low risk	Low risk	Low risk	Unclear timeframe from colonoscopy, MRI, and surgery.
Xia [51]	2024	Low risk	Low risk	Low risk	Low risk	Low risk	Low risk	Low risk	
You [52]	2021	**High risk**	**High risk**	Low risk	Low risk	Low risk	**Unclear risk**	Low risk	Patients with mucinous carcinoma (n = 30) excluded, 89 patients had 3.0 T MRI, and 65 had a separate 3.0 T MRI with different acquisition parameters. Lesion segmentation: obvious areas of necrosis and gas were excluded from subsequent analysis—affecting MRI generalisability.
Yu [53]	2024	Low risk	Low risk	Low risk	Low risk	Low risk	Low risk	Low risk	

## Data Availability

The data underlying this study shall be made available upon reasonable request to the corresponding author.

## Appendix A

### Appendix A.1. Search Strategy

AI Terms:

1. ((((“Machine Learning”[Mesh] OR “Artificial Intelligence”[Mesh] OR “Natural Language

Processing”[Mesh] OR “Neural Networks (Computer)”[Mesh] OR “Support Vector

Machine”[Mesh] OR Machine learning.tw OR Artificial Intelligence.tw OR Naive Bayes.tw OR

bayesian learning.tw OR Neural network.tw OR Neural networks.tw OR Natural language

processing.tw OR support vector *.tw OR random forest *.tw OR deep learning.tw OR deeplearning.tw OR machine intelligence.tw OR computational intelligence.tw OR computer

reasoning.tw OR (Decision Tree Analysis) OR reinforcement learning OR reinforcementlearning OR Boltzmann machine * OR “long short-term memory” OR “gated recurrent unit” OR

“rectified linear unit” OR autoencoder OR backpropagation OR “multilayer perceptron” OR

convnet OR “convolutional learning”)))

AND

Colorectal cancer terms:

2. Colorectal cancer OR colorectal neoplasm OR rectal ca * OR rectal adenocarcinoma OR colorectal adenocarcinoma OR rectal malignancy OR colorectal malignancy

AND

Imaging terms:

3. MRI OR magnetic resonance * OR diffusion magnetic resonance imaging OR diffusion tensor imaging OR fluorine-19 magnetic resonance imaging OR magnetic resonance angiography OR multiparametric magnetic resonance imaging

### Appendix A.2. PICOTS Framework

Population:

Adult patients diagnosed with rectal cancer undergoing preoperative MRI.

Intervention:

Artificial intelligence (AI) based analysis of preoperative MRI for prediction of surgical difficulty (including direct difficulty grading and proxy measures such as T stage, N stage, EMVI, LVI, PNI, ENE, and stapler firings).

Comparator:

Reference standards used in primary studies, including pathological diagnosis, radiologist assessment, and surgeon difficulty scoring.

Outcome:

AI model performance metrics, primarily the area under the receiver operating characteristic curve (AUC), sensitivity, specificity, and accuracy.

Timing:

MRI assessment must be preoperative.

Setting:

Studies conducted in hospital and/or academic settings, without specific limitation. Studies originating from medical, surgical, radiological, or non-healthcare settings were eligible for consideration.

## Appendix B

**Table A1:** PRISMA checklist.

Section and Topic	Item #	Checklist Item	Location Where Item Is Reported
**TITLE**			
Title	1	Identify the report as a systematic review.	1
**ABSTRACT**			
Abstract	2	See the PRISMA 2020 for Abstracts checklist.	1
**INTRODUCTION**			
Rationale	3	Describe the rationale for the review in the context of existing knowledge.	2–3
Objectives	4	Provide an explicit statement of the objective(s) or question(s) the review addresses.	2–3
**METHODS**			
Eligibility criteria	5	Specify the inclusion and exclusion criteria for the review and how studies were grouped for the syntheses.	3
Information sources	6	Specify all databases, registers, websites, organisations, reference lists, and other sources searched or consulted to identify studies. Specify the date when each source was last searched or consulted.	3
Search strategy	7	Present the full search strategies for all databases, registers, and websites, including any filters and limits used.	3 and Appendix A.1
Selection process	8	Specify the methods used to decide whether a study met the inclusion criteria of the review, including how many reviewers screened each record and each report retrieved, whether they worked independently, and, if applicable, details of automation tools used in the process.	3
Data collection process	9	Specify the methods used to collect data from reports, including how many reviewers collected data from each report, whether they worked independently, any processes for obtaining or confirming data from study investigators, and if applicable, details of automation tools used in the process.	3–4
Data items	10a	List and define all outcomes for which data were sought. Specify whether all results that were compatible with each outcome domain in each study were sought (e.g., for all measures, time points, analyses), and if not, the methods used to decide which results to collect.	3–4
	10b	List and define all other variables for which data were sought (e.g., participant and intervention characteristics, funding sources). Describe any assumptions made about any missing or unclear information.	3–4
Study risk of bias assessment	11	Specify the methods used to assess risk of bias in the included studies, including details of the tool(s) used, how many reviewers assessed each study and whether they worked independently, and, if applicable, details of automation tools used in the process.	4
Effect measures	12	Specify for each outcome the effect measure(s) (e.g., risk ratio, mean difference) used in the synthesis or presentation of results.	3–4
Synthesis methods	13a	Describe the processes used to decide which studies were eligible for each synthesis (e.g., tabulating the study intervention characteristics and comparing against the planned groups for each synthesis (item #5)).	3–4
	13b	Describe any methods required to prepare the data for presentation or synthesis, such as handling of missing summary statistics or data conversions.	3–4
	13c	Describe any methods used to tabulate or visually display results of individual studies and syntheses.	4
	13d	Describe any methods used to synthesise results and provide a rationale for the choice(s). If meta-analysis was performed, describe the model(s), method(s) to identify the presence and extent of statistical heterogeneity, and software package(s) used.	4
	13e	Describe any methods used to explore possible causes of heterogeneity among study results (e.g., subgroup analysis, meta-regression).	N/A
	13f	Describe any sensitivity analyses conducted to assess the robustness of the synthesised results.	N/A
Reporting bias assessment	14	Describe any methods used to assess the risk of bias due to missing results in a synthesis (arising from reporting biases).	4
Certainty assessment	15	Describe any methods used to assess certainty (or confidence) in the body of evidence for an outcome.	4
**RESULTS**			
Study selection	16a	Describe the results of the search and selection process, from the number of records identified in the search to the number of studies included in the review, ideally using a flow diagram.	4
	16b	Cite studies that might appear to meet the inclusion criteria but which were excluded, and explain why they were excluded.	4–5 and Figure 1
Study characteristics	17	Cite each included study and present its characteristics.	4–5
Risk of bias in studies	18	Present assessments of the risk of bias for each included study.	14–15, Table 4/Table 5
Results of individual studies	19	For all outcomes, present, for each study: (a) summary statistics for each group (where appropriate) and (b) an effect estimate and its precision (e.g., confidence/credible interval), ideally using structured tables or plots.	4–7, 20–22 and Table 2/Table 3
Results of syntheses	20a	For each synthesis, briefly summarise the characteristics and risk of bias among contributing studies.	21–22 and Table 1, Table 4 and Table 5
	20b	Present results of all statistical syntheses conducted. If a meta-analysis was conducted, present for each the summary estimate and its precision (e.g., confidence/credible interval) and measures of statistical heterogeneity. If comparing groups, describe the direction of the effect.	N/A
	20c	Present results of all investigations of possible causes of heterogeneity among study results.	7–20
	20d	Present results of all sensitivity analyses conducted to assess the robustness of the synthesised results.	N/A
Reporting biases	21	Present assessments of the risk of bias due to missing results (arising from reporting biases) for each synthesis assessed.	21–22 and Table 4/Table 5
Certainty of evidence	22	Present assessments of certainty (or confidence) in the body of evidence for each outcome assessed.	10–14
**DISCUSSION**			
Discussion	23a	Provide a general interpretation of the results in the context of other evidence.	22–24
	23b	Discuss any limitations of the evidence included in the review.	23–24
	23c	Discuss any limitations of the review processes used.	24
	23d	Discuss implications of the results for practice, policy, and future research.	22–24
**OTHER INFORMATION**			
Registration and protocol	24a	Provide registration information for the review, including the register name and registration number, or state that the review was not registered.	3
	24b	Indicate where the review protocol can be accessed, or state that a protocol was not prepared.	3
	24c	Describe and explain any amendments to information provided at registration or in the protocol.	N/A
Support	25	Describe sources of financial or non-financial support for the review and the role of the funders or sponsors in the review.	N/A
Competing interests	26	Declare any competing interests of review authors.	N/A
Availability of data, code, and other materials	27	Report which of the following are publicly available and where they can be found: template data collection forms; data extracted from included studies; data used for all analyses; analytic code; any other materials used in the review.	24

**Table A2:** SWiM Strategy.

SWiM Reporting Item	Item Description	Page in Manuscript Where Item Is Reported	Other *
**Methods**			
1 Grouping studies for synthesis	(1a) Provide a description of, and rationale for, the groups used in the synthesis (e.g., groupings of populations, interventions, outcomes, study design)	3–4	
	(1b) Detail and provide rationale for any changes made subsequent to the protocol in the groups used in the synthesis	N/A	
2 Describe the standardised metric and transformation methods used	Describe the standardised metric for each outcome. Explain why the metric(s) was chosen and describe any methods used to transform the intervention effects, as reported in the study, to the standardised metric, citing any methodological guidance consulted	3–4	
3 Describe the synthesis methods	Describe and justify the methods used to synthesise the effects for each outcome when it was not possible to undertake a meta-analysis of effect estimates	4	
4 Criteria used to prioritise results for summary and synthesis	Where applicable, provide the criteria used, with supporting justification, to select the particular studies, or a particular study, for the main synthesis or to draw conclusions from the synthesis (e.g., based on study design, risk of bias assessments, directness in relation to the review question)	4	
5 Investigation of heterogeneity in reported effects	State the method(s) used to examine heterogeneity in reported effects when it was not possible to undertake a meta-analysis of effect estimates and its extensions to investigate heterogeneity	22–24	
6 Certainty of evidence	Describe the methods used to assess the certainty of the synthesis findings	3–4 and 22–24	
7 Data presentation methods	Describe the graphical and tabular methods used to present the effects (e.g., tables, forest plots, harvest plots)	3–4 and 6–22 And all tables	
	Specify key study characteristics (e.g., study design, risk of bias) used to order the studies, in the text and any tables or graphs, clearly referencing the studies included		
**Results**			
8 Reporting results	For each comparison and outcome, provide a description of the synthesised findings and the certainty of the findings. Describe the result in language that is consistent with the question the synthesis addresses, and indicate which studies contribute to the synthesis	6–22 and all tables	
**Discussion**			
9 Limitations of the synthesis	Report the limitations of the synthesis methods used and/or the groupings used in the synthesis and how these affect the conclusions that can be drawn in relation to the original review question	23–24	

## References

[1] Colorectal Cancer. https://www.who.int/news-room/fact-sheets/detail/colorectal-cancer#:~:text=Regular%20screenings%20are%20crucial%20for,estimated%20to%20have%20occurred%20worldwide.

[2] Siegel R.L., Giaquinto A.N., Jemal A. (2024). Cancer Statistics, 2024. CA Cancer J. Clin..

[3] Khajeh E., Aminizadeh E., Dooghaie Moghadam A., Nikbakhsh R., Goncalves G., Carvalho C., Parvaiz A., Kulu Y., Mehrabi A. (2023). Outcomes of Robot-Assisted Surgery in Rectal Cancer Compared with Open and Laparoscopic Surgery. Cancers.

[4] Park J.S., Lee S.M., Choi G.-S., Park S.Y., Kim H.J., Song S.H., Min B.S., Kim N.K., Kim S.H., Lee K.Y. (2023). Comparison of Laparoscopic versus Robot-Assisted Surgery for Rectal Cancers: The COLRAR Randomized Controlled Trial: The COLRAR Randomized Controlled Trial. Ann. Surg..

[5] Yamamoto T., Kawada K., Kiyasu Y., Itatani Y., Mizuno R., Hida K., Sakai Y. (2020). Prediction of Surgical Difficulty in Minimally Invasive Surgery for Rectal Cancer by Use of MRI Pelvimetry. BJS Open.

[6] Smedh K., Olsson L., Johansson H., Aberg C., Andersson M. (2001). Reduction of Postoperative Morbidity and Mortality in Patients with Rectal Cancer Following the Introduction of a Colorectal Unit: Centralized Rectal Cancer Surgery. Br. J. Surg..

[7] Rajpurkar P., Chen E., Banerjee O., Topol E.J. (2022). AI in Health and Medicine. Nat. Med..

[8] Horvat N., Carlos Tavares Rocha C., Clemente Oliveira B., Petkovska I., Gollub M.J. (2019). MRI of Rectal Cancer: Tumor Staging, Imaging Techniques, and Management. Radiographics.

[9] Page M.J., McKenzie J.E., Bossuyt P.M., Boutron I., Hoffmann T.C., Mulrow C.D., Shamseer L., Tetzlaff J.M., Akl E.A., Brennan S.E. (2021). The PRISMA 2020 Statement: An Updated Guideline for Reporting Systematic Reviews. Rev. Esp. Cardiol..

[10] Krizhevsky A., Sutskever I., Hinton G.E., Pereira F., Burges C.J., Bottou L., Weinberger K.Q. (2012). ImageNet Classification with Deep Convolutional Neural Networks. Advances in Neural Information Processing Systems.

[11] Tejani A.S., Klontzas M.E., Gatti A.A., Mongan J.T., Moy L., Park S.H., Kahn C.E., CLAIM 2024 Update Panel (2024). Checklist for Artificial Intelligence in Medical Imaging (CLAIM): 2024 Update. Radiol. Artif. Intell..

[12] Whiting P.F., Rutjes A.W.S., Westwood M.E., Mallett S., Deeks J.J., Reitsma J.B., Leeflang M.M.G., Sterne J.A.C., Bossuyt P.M.M. (2011). QUADAS-2 Group QUADAS-2: A Revised Tool for the Quality Assessment of Diagnostic Accuracy Studies. Ann. Intern. Med..

[13] Liu X., Rivera S.C., Moher D., Calvert M.J., Denniston A.K., SPIRIT-AI and CONSORT-AI Working Group (2020). Reporting Guidelines for Clinical Trial Reports for Interventions Involving Artificial Intelligence: The CONSORT-AI Extension. BMJ.

[14] Campbell M., McKenzie J.E., Sowden A., Katikireddi S.V., Brennan S.E., Ellis S., Hartmann-Boyce J., Ryan R., Shepperd S., Thomas J. (2020). Synthesis without Meta-Analysis (SWiM) in Systematic Reviews: Reporting Guideline. BMJ.

[15] Cai L., Lambregts D.M.J., Beets G.L., Mass M., Pooch E.H.P., Guérendel C., Beets-Tan R.G.H., Benson S. (2024). An Automated Deep Learning Pipeline for EMVI Classification and Response Prediction of Rectal Cancer Using Baseline MRI: A Multi-Centre Study. NPJ Precis. Oncol..

[16] Ding L., Liu G.-W., Zhao B.-C., Zhou Y.-P., Li S., Zhang Z.-D., Guo Y.-T., Li A.-Q., Lu Y., Yao H.-W. (2019). Artificial Intelligence System of Faster Region-Based Convolutional Neural Network Surpassing Senior Radiologists in Evaluation of Metastatic Lymph Nodes of Rectal Cancer. Chin. Med. J..

[17] Dong X., Ren G., Chen Y., Yong H., Zhang T., Yin Q., Zhang Z., Yuan S., Ge Y., Duan S. (2023). Effects of MRI Radiomics Combined with Clinical Data in Evaluating Lymph Node Metastasis in mrT1-3a Staging Rectal Cancer. Front. Oncol..

[18] Fan L., Wu H., Wu Y., Wu S., Zhao J., Zhu X. (2024). Preoperative Prediction of Rectal Cancer Staging Combining MRI Deep Transfer Learning, Radiomics Features, and Clinical Factors: Accurate Differentiation from Stage T2 to T3. BMC Gastroenterol..

[19] Fang Z., Pu H., Chen X.-L., Yuan Y., Zhang F., Li H. (2023). MRI Radiomics Signature to Predict Lymph Node Metastasis after Neoadjuvant Chemoradiation Therapy in Locally Advanced Rectal Cancer. Abdom. Radiol..

[20] Fang J., Sun W., Wu D., Pang P., Guo X., Yu C., Lu W., Tang G. (2022). Value of Texture Analysis Based on Dynamic Contrast-Enhanced Magnetic Resonance Imaging in Preoperative Assessment of Extramural Venous Invasion in Rectal Cancer. Insights Imaging.

[21] Fu Y., Liu X., Yang Q., Sun J., Xie Y., Zhang Y., Zhang H. (2019). Radiomic Features Based on MRI for Prediction of Lymphovascular Invasion in Rectal Cancer. Chin. J. Acad. Radiol..

[22] Fu Z., Li S., Zang L., Dong F., Cai Z., Ma J. (2023). Predicting Multiple Linear Stapler Firings in Double Stapling Technique with an MRI-Based Deep-Learning Model. Sci. Rep..

[23] Hamabe A., Ishii M., Kamoda R., Sasuga S., Okuya K., Okita K., Akizuki E., Sato Y., Miura R., Onodera K. (2022). Artificial Intelligence-Based Technology for Semi-Automated Segmentation of Rectal Cancer Using High-Resolution MRI. PLoS ONE.

[24] Hamabe A., Takemasa I., Ishii M., Okuya K., Hida K., Nishizaki D., Sumii A., Arizono S., Kohno S., Tokunaga K. (2024). The Potential of an Artificial Intelligence for Diagnosing MRI Images in Rectal Cancer: Multicenter Collaborative Trial. J. Gastroenterol..

[25] Hao Y., Zheng J., Li W., Zhao W., Zheng J., Wang H., Ren J., Zhang G., Zhang J. (2024). Ultra-High B-Value DWI in Rectal Cancer: Image Quality Assessment and Regional Lymph Node Prediction Based on Radiomics. Eur. Radiol..

[26] Hou M., Zhou L., Sun J. (2023). Deep-Learning-Based 3D Super-Resolution MRI Radiomics Model: Superior Predictive Performance in Preoperative T-Staging of Rectal Cancer. Eur. Radiol..

[27] Kasai S., Shiomi A., Kagawa H., Hino H., Manabe S., Yamaoka Y., Chen K., Nanishi K., Kinugasa Y. (2022). The Effectiveness of Machine Learning in Predicting Lateral Lymph Node Metastasis from Lower Rectal Cancer: A Single Center Development and Validation Study. Ann. Gastroenterol. Surg..

[28] Kasai S., Shiomi A., Shimizu H., Aoba M., Kinugasa Y., Miura T., Uehara K., Watanabe J., Kawai K., Ajioka Y. (2024). Risk Factors and Development of Machine Learning Diagnostic Models for Lateral Lymph Node Metastasis in Rectal Cancer: Multicentre Study. BJS Open.

[29] Li J., Zhou Y., Wang P., Zhao H., Wang X., Tang N., Luan K. (2021). Deep Transfer Learning Based on Magnetic Resonance Imaging Can Improve the Diagnosis of Lymph Node Metastasis in Patients with Rectal Cancer. Quant. Imaging Med. Surg..

[30] Li C., Yin J. (2021). Radiomics Based on T2-Weighted Imaging and Apparent Diffusion Coefficient Images for Preoperative Evaluation of Lymph Node Metastasis in Rectal Cancer Patients. Front. Oncol..

[31] Li H., Chai L., Pu H., Yin L.-L., Li M., Zhang X., Liu Y.-S., Pang M.-H., Lu T. (2024). T2WI-Based MRI Radiomics for the Prediction of Preoperative Extranodal Extension and Prognosis in Resectable Rectal Cancer. Insights Imaging.

[32] Lin X., Jiang H., Zhao S., Hu H., Jiang H., Li J., Jia F. (2024). MRI-Based Radiomics Model for Preoperative Prediction of Extramural Venous Invasion of Rectal Adenocarcinoma. Acta Radiol..

[33] Liu L., Liu Y., Xu L., Li Z., Lv H., Dong N., Li W., Yang Z., Wang Z., Jin E. (2017). Application of Texture Analysis Based on Apparent Diffusion Coefficient Maps in Discriminating Different Stages of Rectal Cancer. J. Magn. Reson. Imaging.

[34] Liu S., Yu X., Yang S., Hu P., Hu Y., Chen X., Li Y., Zhang Z., Li C., Lu Q. (2021). Machine Learning-Based Radiomics Nomogram for Detecting Extramural Venous Invasion in Rectal Cancer. Front. Oncol..

[35] Lu Y., Yu Q., Gao Y., Zhou Y., Liu G., Dong Q., Ma J., Ding L., Yao H., Zhang Z. (2018). Identification of Metastatic Lymph Nodes in MR Imaging with Faster Region-Based Convolutional Neural Networks. Cancer Res..

[36] Lu H., Yuan Y., Zhou Z., Ma X., Shen F., Xia Y., Lu J. (2021). Assessment of MRI-Based Radiomics in Preoperative T Staging of Rectal Cancer: Comparison between Minimum and Maximum Delineation Methods. Biomed Res. Int..

[37] Ma X., Shen F., Jia Y., Xia Y., Li Q., Lu J. (2019). MRI-Based Radiomics of Rectal Cancer: Preoperative Assessment of the Pathological Features. BMC Med. Imaging.

[38] Ma S., Lu H., Jing G., Li Z., Zhang Q., Ma X., Chen F., Shao C., Lu Y., Wang H. (2023). Deep Learning-Based Clinical-Radiomics Nomogram for Preoperative Prediction of Lymph Node Metastasis in Patients with Rectal Cancer: A Two-Center Study. Front. Med..

[39] Niu Y., Yu X., Wen L., Bi F., Jian L., Liu S., Yang Y., Zhang Y., Lu Q. (2023). Comparison of Preoperative CT- and MRI-Based Multiparametric Radiomics in the Prediction of Lymph Node Metastasis in Rectal Cancer. Front. Oncol..

[40] Shu Z., Mao D., Song Q., Xu Y., Pang P., Zhang Y. (2022). Multiparameter MRI-Based Radiomics for Preoperative Prediction of Extramural Venous Invasion in Rectal Cancer. Eur. Radiol..

[41] Sun Z., Hou W., Liu W., Liu J., Li K., Wu B., Lin G., Xue H., Pan J., Xiao Y. (2023). Establishment of Surgical Difficulty Grading System and Application of MRI-Based Artificial Intelligence to Stratify Difficulty in Laparoscopic Rectal Surgery. Bioengineering.

[42] Sun Y., Lu Z., Yang H., Jiang P., Zhang Z., Liu J., Zhou Y., Li P., Zeng Q., Long Y. (2024). Prediction of Lateral Lymph Node Metastasis in Rectal Cancer Patients Based on MRI Using Clinical, Deep Transfer Learning, Radiomic, and Fusion Models. Front. Oncol..

[43] Tian C., Ma X., Lu H., Wang Q., Shao C., Yuan Y., Shen F. (2023). Deep Learning Models for Preoperative T-Stage Assessment in Rectal Cancer Using MRI: Exploring the Impact of Rectal Filling. Front. Med..

[44] Wan L., Hu J., Chen S., Zhao R., Peng W., Liu Y., Hu S., Zou S., Wang S., Zhao X. (2023). Prediction of Lymph Node Metastasis in Stage T1-2 Rectal Cancers with MRI-Based Deep Learning. Eur. Radiol..

[45] Wang Y., Chen A., Wang K., Zhao Y., Du X., Chen Y., Lv L., Huang Y., Ma Y. (2024). Predictive Study of Machine Learning-Based Multiparametric MRI Radiomics Nomogram for Perineural Invasion in Rectal Cancer: A Pilot Study. J. Imaging Inform. Med..

[46] Wei Q., Chen L., Hou X., Lin Y., Xie R., Yu X., Zhang H., Wen Z., Wu Y., Liu X. (2024). Multiparametric MRI-Based Radiomic Model for Predicting Lymph Node Metastasis after Neoadjuvant Chemoradiotherapy in Locally Advanced Rectal Cancer. Insights Imaging.

[47] Wei Q., Yuan W., Jia Z., Chen J., Li L., Yan Z., Liao Y., Mao L., Hu S., Liu X. (2023). Preoperative MR Radiomics Based on High-Resolution T2-Weighted Images and Amide Proton Transfer-Weighted Imaging for Predicting Lymph Node Metastasis in Rectal Adenocarcinoma. Abdom. Radiol..

[48] Wei Y., Wang H., Chen Z., Zhu Y., Li Y., Lu B., Pan K., Wen C., Cao G., He Y. (2024). Deep Learning-Based Multiparametric MRI Model for Preoperative T-Stage in Rectal Cancer. J. Magn. Reson. Imaging.

[49] Wong C., Liu T., Zhang C., Li M., Zhang H., Wang Q., Fu Y. (2024). Preoperative Detection of Lymphovascular Invasion in Rectal Cancer Using Intravoxel Incoherent Motion Imaging Based on Radiomics. Med. Phys..

[50] Wu Q.-Y., Liu S.-L., Sun P., Li Y., Liu G.-W., Liu S.-S., Hu J.-L., Niu T.-Y., Lu Y. (2021). Establishment and Clinical Application Value of an Automatic Diagnosis Platform for Rectal Cancer T-Staging Based on a Deep Neural Network. Chin. Med. J..

[51] Xia W., Li D., He W., Pickhardt P.J., Jian J., Zhang R., Zhang J., Song R., Tong T., Yang X. (2024). Multicenter Evaluation of a Weakly supervISed Deep Learning Model for Lymph Node Diagnosis in Rectal Cancer at MRI. Radiol. Artif. Intell..

[52] You J., Yin J. (2021). Performances of Whole Tumor Texture Analysis Based on MRI: Predicting Preoperative T Stage of Rectal Carcinomas. Front. Oncol..

[53] Yu M., Yuan Z., Li R., Shi B., Wan D., Dong X. (2024). Interpretable Machine Learning Model to Predict Surgical Difficulty in Laparoscopic Resection for Rectal Cancer. Front. Oncol..

[54] Liu X., Yang Q., Zhang C., Sun J., He K., Xie Y., Zhang Y., Fu Y., Zhang H. (2020). Multiregional-Based Magnetic Resonance Imaging Radiomics Combined With Clinical Data Improves Efficacy in Predicting Lymph Node Metastasis of Rectal Cancer. Front. Oncol..

[55] Escal L., Nougaret S., Guiu B., Bertrand M.M., de Forges H., Tetreau R., Thézenas S., Rouanet P. (2018). MRI-Based Score to Predict Surgical Difficulty in Patients with Rectal Cancer. Br. J. Surg..

[56] Dindo D., Demartines N., Clavien P.-A. (2004). Classification of Surgical Complications: A New Proposal with Evaluation in a Cohort of 6336 Patients and Results of a Survey. Ann. Surg..

[57] Kotu V., Deshpande B. (2019). Data Science Process. Data Science.

[58] Lambregts D.M.J., Bogveradze N., Blomqvist L.K., Fokas E., Garcia-Aguilar J., Glimelius B., Gollub M.J., Konishi T., Marijnen C.A.M., Nagtegaal I.D. (2022). Current Controversies in TNM for the Radiological Staging of Rectal Cancer and How to Deal with Them: Results of a Global Online Survey and Multidisciplinary Expert Consensus. Eur. Radiol..

[59] Xynos E., Tekkis P., Gouvas N., Vini L., Chrysou E., Tzardi M., Vassiliou V., Boukovinas I., Agalianos C., Androulakis N. (2016). Clinical Practice Guidelines for the Surgical Treatment of Rectal Cancer: A Consensus Statement of the Hellenic Society of Medical Oncologists (HeSMO). Ann. Gastroenterol..

[60] Chand M., Siddiqui M.R.S., Swift I., Brown G. (2016). Systematic Review of Prognostic Importance of Extramural Venous Invasion in Rectal Cancer. World J. Gastroenterol..

[61] Peacock O., Manisundaram N., Dibrito S.R., Kim Y., Hu C.-Y., Bednarski B.K., Konishi T., Stanietzky N., Vikram R., Kaur H. (2022). Magnetic Resonance Imaging Directed Surgical Decision Making for Lateral Pelvic Lymph Node Dissection in Rectal Cancer after Total Neoadjuvant Therapy (TNT). Ann. Surg..

[62] Sun Q., Liu T., Liu P., Luo J., Zhang N., Lu K., Ju H., Zhu Y., Wu W., Zhang L. (2019). Perineural and Lymphovascular Invasion Predicts for Poor Prognosis in Locally Advanced Rectal Cancer after Neoadjuvant Chemoradiotherapy and Surgery. J. Cancer.

[63] Braunschmid T., Hartig N., Baumann L., Dauser B., Herbst F. (2017). Influence of Multiple Stapler Firings Used for Rectal Division on Colorectal Anastomotic Leak Rate. Surg. Endosc..

[64] Balciscueta Z., Uribe N., Caubet L., López M., Torrijo I., Tabet J., Martín M.C. (2020). Impact of the Number of Stapler Firings on Anastomotic Leakage in Laparoscopic Rectal Surgery: A Systematic Review and Meta-Analysis. Tech. Coloproctol..

[65] de Hond A.A.H., Steyerberg E.W., van Calster B. (2022). Interpreting Area under the Receiver Operating Characteristic Curve. Lancet Digit. Health.

[66] Lam K., Clarke J., Purkayastha S., Kinross J.M. (2021). Uptake and Accessibility of Surgical Robotics in England. Int. J. Med. Robot..

[67] Ying X. (2019). An Overview of Overfitting and Its Solutions. J. Phys. Conf. Ser..

[68] Yang Y., Wang H.-Y., Chen Y.-K., Chen J.-J., Song C., Gu J. (2020). Current Status of Surgical Treatment of Rectal Cancer in China. Chin. Med. J..

[69] Center for Devices, Radiological Health Overview of Device Regulation. https://www.fda.gov/medical-devices/device-advice-comprehensive-regulatory-assistance/overview-device-regulation.

[70] Provisions for Medical Device Registration and Filing. https://english.nmpa.gov.cn/2024-06/05/c_993242.htm.

[71] Regulation—2017/745—EN—Medical Device Regulation—EUR-Lex. https://eur-lex.europa.eu/legal-content/EN/TXT/?uri=celex%3A32017R0745.

[72] EUR-Lex—L:2017:117:TOC—EN—EUR-Lex. https://eur-lex.europa.eu/legal-content/EN/TXT/?uri=OJ:L:2017:117:TOC.

[73] Longoni C., Bonezzi A., Morewedge C.K. (2019). Resistance to Medical Artificial Intelligence. J. Consum. Res..

[74] Radiology AI Health Register of EU CE Marked Devices. https://radiology.healthairegister.com/.

[75] AI Central Database of FDA Approved Radiology AI. https://aicentral.acrdsi.org/.

[76] Obuchowicz R., Lasek J., Wodziński M., Piórkowski A., Strzelecki M., Nurzynska K. (2025). Artificial Intelligence-Empowered Radiology-Current Status and Critical Review. Diagnostics.

[77] Kolbinger F.R., Veldhuizen G.P., Zhu J., Truhn D., Kather J.N. (2024). Reporting Guidelines in Medical Artificial Intelligence: A Systematic Review and Meta-Analysis. Commun. Med..

[78] Lekadir K., Frangi A.F., Porras A.R., Glocker B., Cintas C., Langlotz C.P., Weicken E., Asselbergs F.W., Prior F., Collins G.S. (2025). FUTURE-AI: International Consensus Guideline for Trustworthy and Deployable Artificial Intelligence in Healthcare. BMJ.

[79] Lambregts D.M.J., Vandecaveye V., Barbaro B., Bakers F.C.H., Lambrecht M., Maas M., Haustermans K., Valentini V., Beets G.L., Beets-Tan R.G.H. (2011). Diffusion-Weighted MRI for Selection of Complete Responders after Chemoradiation for Locally Advanced Rectal Cancer: A Multicenter Study. Ann. Surg. Oncol..

